# Hippocampal morphometry is altered in infants with congenital heart disease

**DOI:** 10.1093/braincomms/fcag060

**Published:** 2026-02-26

**Authors:** Barat Gal-Er, Alexandra F Bonthrone, Emily S Nichols, Andrew T M Chew, Daniel Cromb, Chiara Casella, Alexia Egloff, Kuberan Pushparajah, John Simpson, Mary A Rutherford, A David Edwards, Joseph V Hajnal, Chiara Nosarti, Jonathan O’Muircheartaigh, Emma G Duerden, Serena J Counsell

**Affiliations:** Centre for the Developing Brain, Research Department of Early Life Imaging, School of Biomedical Engineering and Imaging Sciences, King’s College London, London SE1 7EH, UK; Centre for the Developing Brain, Research Department of Early Life Imaging, School of Biomedical Engineering and Imaging Sciences, King’s College London, London SE1 7EH, UK; Department of Applied Psychology, Faculty of Education, Western University, London, Ontario, Canada N6G 1G7; Western Institute for Neuroscience, Western University, London, Ontario, Canada N6A 3K7; Centre for the Developing Brain, Research Department of Early Life Imaging, School of Biomedical Engineering and Imaging Sciences, King’s College London, London SE1 7EH, UK; Centre for the Developing Brain, Research Department of Early Life Imaging, School of Biomedical Engineering and Imaging Sciences, King’s College London, London SE1 7EH, UK; Centre for the Developing Brain, Research Department of Early Life Imaging, School of Biomedical Engineering and Imaging Sciences, King’s College London, London SE1 7EH, UK; Department for Forensic and Neurodevelopmental Sciences, Institute of Psychiatry, Psychology and Neuroscience, King’s College London, London SE5 8AB, UK; Centre for the Developing Brain, Research Department of Early Life Imaging, School of Biomedical Engineering and Imaging Sciences, King’s College London, London SE1 7EH, UK; Department of Cardiovascular Imaging, School of Biomedical Engineering and Imaging Sciences, King’s College London, London SE1 7EH, UK; Department of Fetal and Paediatric Cardiology, Evelina London Children’s Hospital, London SE1 7EH, UK; Department of Cardiovascular Imaging, School of Biomedical Engineering and Imaging Sciences, King’s College London, London SE1 7EH, UK; Department of Fetal and Paediatric Cardiology, Evelina London Children’s Hospital, London SE1 7EH, UK; Centre for the Developing Brain, Research Department of Early Life Imaging, School of Biomedical Engineering and Imaging Sciences, King’s College London, London SE1 7EH, UK; Centre for the Developing Brain, Research Department of Early Life Imaging, School of Biomedical Engineering and Imaging Sciences, King’s College London, London SE1 7EH, UK; Centre for the Developing Brain, Research Department of Early Life Imaging, School of Biomedical Engineering and Imaging Sciences, King’s College London, London SE1 7EH, UK; Research Department of Imaging Physics & Engineering, School of Biomedical Engineering and Imaging Sciences, King's College London, London SE1 7EH, UK; Centre for the Developing Brain, Research Department of Early Life Imaging, School of Biomedical Engineering and Imaging Sciences, King’s College London, London SE1 7EH, UK; Department of Child and Adolescent Psychiatry, Institute of Psychiatry, Psychology and Neuroscience, King's College London, London SE5 8AB, UK; Centre for the Developing Brain, Research Department of Early Life Imaging, School of Biomedical Engineering and Imaging Sciences, King’s College London, London SE1 7EH, UK; Department for Forensic and Neurodevelopmental Sciences, Institute of Psychiatry, Psychology and Neuroscience, King’s College London, London SE5 8AB, UK; Department of Applied Psychology, Faculty of Education, Western University, London, Ontario, Canada N6G 1G7; Western Institute for Neuroscience, Western University, London, Ontario, Canada N6A 3K7; Centre for the Developing Brain, Research Department of Early Life Imaging, School of Biomedical Engineering and Imaging Sciences, King’s College London, London SE1 7EH, UK

**Keywords:** congenital heart disease, hippocampus, MRI, morphometry, cerebral oxygen delivery

## Abstract

Congenital heart disease (CHD) is associated with impaired early brain development and an increased risk of adverse neurodevelopmental outcomes. Previous studies have reported smaller hippocampal volumes in infants, children and adolescents with CHD. However, it is unclear whether specific subfields are differentially sensitive as hippocampal subfield morphometry has not been assessed in this population. The aims of this study were to test the hypothesis that infants with CHD deviate from typical hippocampal morphometry, using a normative modelling approach with reference data from 217 typically developing infants, and characterize the relationship between hippocampal morphometric measures and both cerebral oxygen delivery and neurodevelopmental outcomes in infants with CHD. Infants with CHD [60 preoperative and 29 postoperative, gestational age at birth median (range) 38.43 (36.71–40.57) weeks, postmenstrual age at scan 39.86 (37.14–45.71) weeks] and typically developing infants underwent brain MRI on a 3T scanner, and T_2_-weighted and inversion recovery T_1_-weighted imaging were acquired. Phase-contrast angiography was acquired in 53 infants with CHD before surgery, and cerebral oxygen delivery was calculated. Cognitive and motor abilities were assessed at 22 months (*N* = 52) using the Bayley Scales of Infant and Toddler Development-Third Edition. Volumes of the whole hippocampus, subiculum, cornu ammonis 1–4, dentate gyrus, and stratum radiatum lacunosum and moleculare were obtained by segmenting T_1_-weighted MRI data using a U-net model trained on infant data using HippUnfold. Normative curves were generated from the typically developing infants using two models: one for relative hippocampal volumes and one for hippocampal gyrification. Models accounted for the infant’s postmenstrual age at scan, postnatal age at scan and sex. The hippocampal gyrification model also accounted for hippocampal subfield volume. Z-scores representing the degree of positive or negative deviation from the normative mean were generated for infants with CHD. Relative volume z-scores were reduced for the bilateral cornu ammonis 4 and dentate gyrus (median Z-scores −0.45 to −0.82) and were increased for the left subiculum (mean Z-scores: preoperative 0.44; postoperative 0.51) in infants with CHD. Hippocampal gyrification was reduced in the bilateral subiculum, cornu ammonis 1–4 and dentate gyrus both pre- and postoperatively (mean Z-scores, −0.50 to −1.33). There was no significant relationship between preoperative hippocampal morphometric Z-scores and cerebral oxygen delivery or neurodevelopmental outcomes. Our findings suggest that hippocampal morphometry is altered in infants with CHD; however these effects are not uniform across the hippocampus.

## Teaser

Gal-Er *et al*. report that infants with congenital heart disease show altered hippocampal subfield morphometry, with reduced relative volumes of the cornu ammonis 4 and dentate gyrus, increased relative volume of the left subiculum and widespread reductions in gyrification. These alterations were not associated with cerebral oxygen delivery or neurodevelopment.

## Graphical Abstract

**Graphical Abstract:**
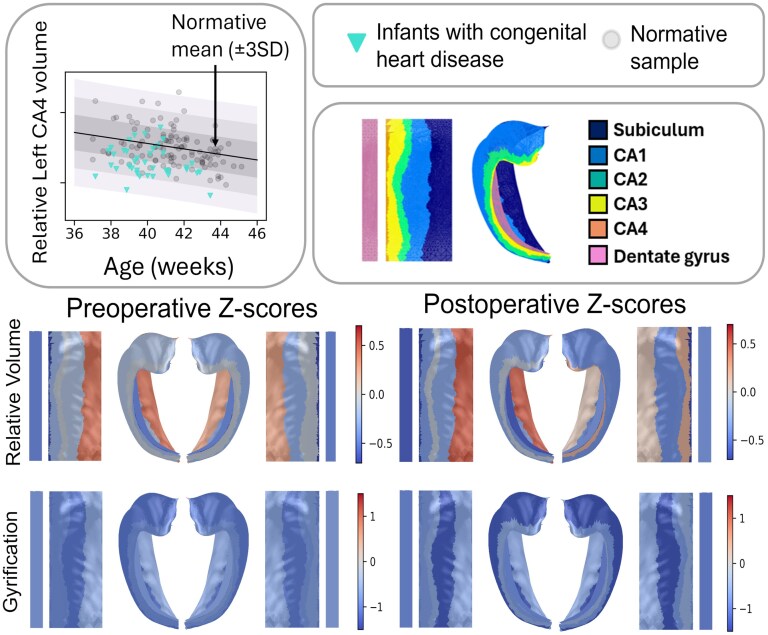

## Introduction

Congenital heart disease (CHD) is the most common congenital malformation, with a prevalence of approximately 8 per 1000 births.^1^ CHD is associated with altered early brain development^2-7^ and subsequent neurodevelopmental impairments.^8-11^ Previous MRI studies have shown that hippocampal development is altered in this population, with smaller hippocampal volumes reported in fetuses,^12^ infants,^5,13^ children,^14-16^ and adolescents.^17-19^ Furthermore, smaller hippocampal volumes have been associated with lower IQ and poorer working memory in individuals with CHD.^14,15,17-19^

In previous neonatal neuroimaging studies, the hippocampus has been modelled as a single volume.^5,13^ However, this structure has a complex composition, including multiple subfields, each with distinct cytoarchitectural and functional properties.^20^ This complexity motivates a more anatomically specific approach for assessing hippocampal subfield morphometry. In addition, the hippocampus has a folded archicortical mantle that is contiguous with the cerebral cortex,^20^ and this folding, or gyrification, has been shown to vary considerably between individuals.^21^ To date, hippocampal gyrification in CHD has not been assessed.

Previous studies investigating hippocampal morphometry in individuals with CHD have been performed at the group level using case-control comparisons, which assume homogenous effects across the population. Normative modelling techniques map data from individual infants to a reference distribution derived from a control cohort, enabling the quantification of deviations from typical development at the individual level. This approach has been used previously to investigate brain volumes^7,22^ and cortical folding^23^ in infants with CHD. However, normative modelling has not yet been applied to study hippocampal subfield volume and gyrification in this population.

The aims of this study were (i) to test the hypothesis that hippocampal morphometry, as measured by hippocampal subfield volumes and gyrification, in infants with CHD deviates from the typical trajectory of hippocampal morphometry, as defined using a large cohort of typically developing infants; (ii) to assess the relationship between hippocampal morphometric measures and preoperative cerebral oxygen delivery in infants with CHD; and (iii) to investigate whether hippocampal morphometry is associated with early childhood neurodevelopmental outcomes in infants with CHD.

## Materials and methods

### Ethical approval

Research Ethics Committee approval was obtained for this study [Congenital Heart Imaging Programme (CHiP): 07/H0707/105 and 21/WA/0075; Developing Human Connectome Project (dHCP): 14/LO/1169]. Informed written parental consent was obtained before collection of MRI and neurodevelopmental assessment data.

### CHD participants

#### Recruitment and CHD categorization

Infants with CHD were prospectively recruited from the Neonatal and Paediatric Intensive Care Units at the Evelina London Children's Hospital between 2020 and 2023 as part of the CHiP study.

Participants were recruited if they had critical or serious CHD,^24^ as described previously.^7,23^ Each infant was also allocated to one of the three diagnostic CHD categories based on the haemodynamic impact of the underlying cardiac diagnosis, using the sequential segmental approach: (i) abnormal streaming of the blood, (ii) left-sided cardiac lesions or (iii) right-sided cardiac lesions.^25^

#### Inclusion criteria

Infants with CHD were eligible for inclusion if they had a diagnosis of critical or serious CHD, if they did not undergo any other neonatal surgery before their cardiac surgery or intervention, if they were born ≥36.00 gestational weeks and if they were scanned between 37 and 46 postmenstrual weeks (number of weeks of gestation at birth plus postnatal age in weeks) and had good-quality T_1_–weighted MRI data.

### Typically developing participants

#### Inclusion criteria

To model typical development, we used the open access Developing Human Connectome Project (dHCP; http://developingconnectome.org/) data as our reference cohort, available at https://nda.nih.gov/edit_collection.html?id=3955. Healthy control infants recruited for the CHiP study were also included if they met the inclusion criteria. Infants were included if they had good-quality T_1_–weighted MRI data, were born ≥36.00 gestational weeks and scanned between 37 and 46 postmenstrual weeks and if no major brain lesions (arterial ischaemic stroke, parenchymal haemorrhage, >10 foci of white matter injury) were reported on their neuroimaging after review by two perinatal neuroradiologists. Infants with incidental findings [*N* = 98] (subdural haemorrhage, isolated subependymal cysts, mild white matter injury) with unlikely significance for clinical outcome or analysis were included. Infants were also eligible for inclusion if they had no severe developmental impairments at 18 months of age. Infants with cognitive and motor scores below 70 (two standard deviations below mean) at 18 months [as assessed using the Bayley Scales of Infant and Toddler Development, Third Edition (Bayley-III)]^26^ were excluded. Infants were also excluded if they had a psychiatric family history or if they had prenatal exposure to COVID during pregnancy.

#### Magnetic resonance imaging

MRI was performed on a Philips Achieva 3 Tesla system (Best, NL) situated on the Neonatal Unit at St Thomas’ Hospital, London, UK, as described previously.^23^

Infants were scanned with a 32-channel neonatal head coil and neonatal positioning system (Rapid Biomedical GmbH, Rimpar DE).^27^ Two T_1_-weighted images were acquired using an Inversion Recovery Turbo Spin Echo (TSE) sequence [repetition time/inversion time/echo time = 4795/1740/8.7 ms; voxel size = 0.8 × 0.8 × 1.6 mm, SENSE factor 2.2 (axial) and 2.6 (sagittal)]. T2-weighted images were acquired using a multi-slice TSE sequence [repetition time/echo time = 12 000/156 ms; voxel size = 0.8 × 0.8 × 1.6 mm, SENSE factor 2.0 (axial) and 2.5 (sagittal)]. T_1_-weighted and T_2_-weighted volumes were reconstructed using a dedicated algorithm to correct motion and integrate data from both acquired stacks (reconstructed voxel size = 0.5 mm^3^).^28,29^

#### Cerebral blood flow and cerebral oxygen delivery in CHD infants

For infants with CHD, cerebral blood flow (CBF) was quantified from phase-contrast angiography using previously published methods.^3^ Haemoglobin (Hb) levels were measured in CHD participants as part of routine clinical care at a median (range) of 2 (0–10) days prior to the scan. Pre-ductal arterial oxygen saturation (SaO_2_) was measured at the time of the scan using a pulse-oximeter applied to the right hand. Cerebral oxygen delivery (CDO_2_) was calculated using the following formula^30^:


$$\mathrm{CD}O_{2}\;(\mathrm{ml}\;O_{2}\;\mathrm{mi}n^{\text{-}1})=\mathrm{Sa}O_{2}\;x\;[\mathrm{Hb}]\;(\mathrm{gd}l^{\text{-}1})\;x\;1.36\;x\;[\mathrm{CBF}]\;(\mathrm{ml}\;\mathrm{mi}n^{\text{-}1})$$

where 1.36 is the amount of oxygen bound per gram of Hb at one atmosphere pressure (Hüfner’s constant).

#### Image preprocessing

T_2_-weighted images were processed using the dHCP structural pipeline.^31^ Images underwent bias field correction and brain extraction before being segmented into eight tissue classes [cortical grey matter (GM), white matter (WM), deep grey matter (dGM), cerebellum, brainstem, hippocampus and amygdala, ventricles and extracerebral CSF] with an automatic neonatal-specific segmentation algorithm.^31^ Total tissue volume (TTV) was calculated by summing cortical GM, WM, cerebellum, brainstem, dGM and hippocampus and amygdala volumes.

#### Hippocampal segmentation

Surface-based segmentation and unfolding of the hippocampus and dentate gyrus (DG) was performed with HippUnfold (https://hippunfold.khanlab.ca/en/latest/) on T_1_-weighted imaging data using a U-net model trained on neonatal data.^32^ Volumes of the subiculum, cornu ammonis (CA) 1,2,3,4, DG, stratum radiatum lacunosum and moleculare (SRLM) were extracted. Total left and right hippocampal volumes were calculated by summing hippocampal subfield volumes. We calculated total hippocampus and hippocampal subfield volumes in absolute (mm^3^) and relative (%) values. Relative volumes were calculated by dividing each regional and total hippocampal volume by TTV. Relative volumes were used in the analysis to ensure results were not driven by individual differences in the global brain size.

#### Visual inspection of hippocampal segmentations

**Figure 1:**
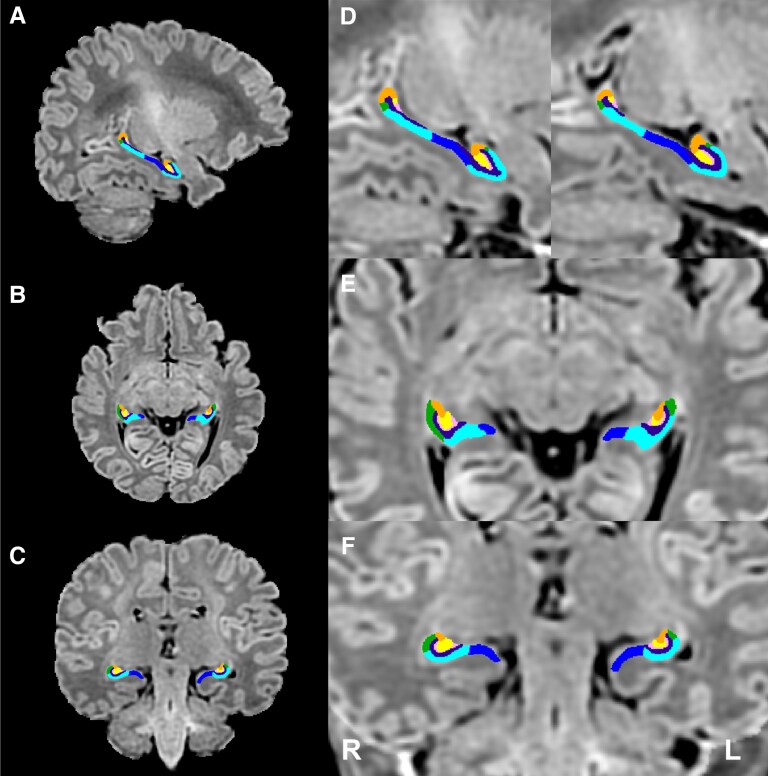
**Hippocampal subfield segmentation**. An example of a hippocampal subfield segmentation in an infant with CHD scanned preoperatively (postmenstrual age at scan: 40.71 weeks, male, TGA) is shown in the (A, D) sagittal, (B, E) axial and (C, F) coronal planes. Hippocampal subfields: subiculum (dark blue), CA1 (light blue), CA2 (green), CA3 (orange), CA4 (yellow), DG, (pink), SRLM (purple). Abbreviations: CA, cornus ammonis; CHD, congenital heart disease; DG, dentate gyrus; L, left; R, right; SRLM, stratum radiatum lacunosum and moleculare; TGA, transposition of the great arteries.

HippUnfold automatically generates quality control (QC) files, including a dice overlap score by comparing the dice overlap of the generated hippocampal segmentation with a conventional deformable registration.^20^ After applying deformable fast B-spline registration to the template, the overlap between hippocampal tissue segmentations and template hippocampal masks was examined. As per the HippUnfold^20^ documentation (https://hippunfold.readthedocs.io/en/latest/index.html), we excluded participants with a dice overlap score of less than 0.7 (*N* = 12, 5 control, 7 CHD). Following this, all hippocampal segmentations were visually reviewed, and those where HippUnfold had failed to generate anatomically accurate hippocampal segmentations were excluded from further analysis (*N* = 46; 38 controls, 8 CHD). An example of a hippocampal segmentation in an infant with CHD is shown in Fig. 1.

#### Hippocampal gyrification

To generate hippocampal surfaces, HippUnfold uses hippocampal boundary regions to establish a subject-specific, continuous intrinsic coordinate system within the hippocampal GM. These coordinates are used to generate non-linear transformations between the native space and standard unfolded hippocampal space. Using the non-linear transformations, standard template unfolded meshes are transformed to each subjects’ hippocampus, to obtain surface meshes in the native space. Vertices in the meshes were assigned to CA1–4, subiculum or DG subfields. Hippocampal gyrification was calculated as the ratio of native space hippocampal surface area over unfolded space hippocampal surface area, where the surface area is calculated at each vertex as the average of areas of connected triangles.^20,33^ For more details, refer to DeKraker *et al*.^20^ Vertex gyrification values for the subiculum, CA1, CA2, CA3, CA4 and DG were averaged separately for the left and right subfields.

#### Modelling typical hippocampal morphometry

Normative modelling of typical hippocampal development was conducted using Gaussian Process Regression (GPR), as described previously^7,22,23,34^ (https://github.com/ralidimitrova/GPR_NeoVols). GPR was used to generate two normative models for each region: one model for relative volume (total hippocampus, subiculum, CA1, CA2, CA3, CA4, DG and SRLM) and one model for gyrification (subiculum, CA1, CA2, CA3, CA4 and DG). Both models accounted for the infant’s postmenstrual age at scan, postnatal age at scan and sex. The gyrification model also accounted for volume of the same structure. These GPR models were then used to assess deviation from the normative development trajectory for each individual infant with CHD. The difference between predicted and observed values, normalized by the predictive confidence, represents the deviation of a data point from the expected mean. For each model, a Z-score quantifying the degree of deviation for the hippocampal morphometric measure was calculated for each infant with CHD.

#### Neurodevelopmental outcomes

Infants with CHD were invited to attend a follow-up neurodevelopmental assessment at 22 months of age. Infants completed the Bayley-III, administered by an experienced developmental paediatrician. Cognitive and motor composite scores were evaluated. (As many of the children in this study come from households where more than one language is spoken and English is often not their first language, we elected not to study the association between language scores and hippocampal morphometry).

#### Socioeconomic status

Index of multiple deprivation (IMD) was calculated using the mother’s postcode at birth for all infants. IMD is a composite measure of socioeconomic status in England encompassing factors such as income, employment, education, health and crime (https://imd-by-postcode.opendatacommunities.org/imd/2019). IMD was calculated from the 2019 data release and reported as quintiles. IMD was not available for one infant with CHD and for two typically developing infants.

### Statistical analysis

A Shapiro–Wilk test was used to assess normality. For normally distributed data, results are reported as mean and standard deviation (SD) and two-tailed t-tests were performed to compare Z-scores between typically developing infants and infants with CHD for all hippocampal morphometric measures, separately for preoperative and postoperative CHD. For non-normally distributed data, results are reported as median and interquartile range (IQR), and the Mann–Whitney U test was performed to compare Z-scores between typically developing infants and infants with CHD for all hippocampal morphometric measures, separately for preoperative and postoperative CHD.

A post hoc analysis was undertaken using two-tailed t-tests for normally distributed data and Mann–Whitney U tests for non-normally distributed data, to compare hippocampal morphometric Z-scores between typically developing infants and each of the CHD categories (abnormal streaming of blood, left-sided cardiac lesions and right-sided cardiac lesions), separately for preoperative and postoperative CHD. Our sample of infants with right-sided cardiac lesions scanned postoperatively was small (*N* = 3), and we were therefore unable to compare hippocampal morphometry in these infants with typically developing infants.

Two-tailed t-tests for normally distributed data and Mann–Whitney U tests for non-normally distributed data were used to compare hippocampal morphometric Z-scores between infants with cyanotic and acyanotic CHD, separately for preoperative and postoperative CHD.

Pearson’s correlation coefficient was calculated to determine the direction and strength of the relationship between hippocampal morphometric Z-scores and CDO_2_ for normally distributed data and Spearman’s Rank correlation was calculated for skewed data.

Partial Spearman’s Rank correlations were used to characterize the relationship between preoperative hippocampal morphometric Z-scores in infants with CHD and neurodevelopmental outcome scores, with IMD rank included as a covariate. Differences in IMD quintiles between groups were assessed with χ^2^ or Fisher’s exact test. Spearman’s correlation coefficient was calculated to determine the direction and strength of the relationship between hippocampal morphometric Z-scores and IMD. Differences in demographics between infants with and without neurodevelopmental follow-up were assessed with Mann–Witney U test or χ^2^.

Benjamini–Hochberg False Discovery Rate^35^ (FDR) was applied to correct for multiple comparisons (reported as pFDR). Statistical analyses were performed using Jupyter Notebook and R v4.2.2.

### Sensitivity analyses

In order to assess whether the presence of infants with genetic syndromes confounded the results, analyses were repeated after removing infants with genetic syndromes (*N* = 4).

## Results

### Participant characteristics

Sixty-five participants with CHD met the inclusion criteria (60 preoperative scans; 29 postoperative scans) and were analysed. For the typically developing cohort, data from 212 participants from the dHCP study and 5 control participants from the CHiP study met the inclusion criteria. Demographics for all participants are summarized in Table 1. Four infants with CHD had genetic or syndromic diagnoses confirmed postnatally: three with 22q11.2 deletion (ToF, *N* = 1; truncus arteriosus, *N* = 1; IAA, *N* = 1) and one with a RASopathy (CoA).

**Table 1:** Participant demographics and clinical characteristics

	Controls (*N* = 217)	Preoperative CHD (*N* = 60)	Postoperative CHD (*N* = 29)
Gestational age at birth [weeks], median (range)	40.14 (36.14–42.29)	38.57 (36.71–40.57)	38.43 (36.85–39.72)
Postmenstrual age at scan [weeks], median (range)	41.14 (37.0–45.14)	39.29 (37.14–42.43)	41.29 (38.86–45.71)
Time from birth to scan [days], median (range)	4 (0–61)	4 (1–37)	18 (9–62)
Male, *N* (%)	106 (49)	34 (57)	16 (55)
Birth weight [grams], mean (±SD)	3352 (±500)	3146 (±589)	3141 (±591)
CDO_2_ [ml O_2_ min^−1^], mean (±SD), (*N* = 53)	-	1725 (±384)	-
Primary cardiac defect, *N* (%)			
Abnormal streaming			
Transposition of the great arteries		20 (33)	15 (52)
Double outlet right ventricle		3 (5)	0
Total anomalous pulmonary venous drainage		3 (5)	0
Truncus arteriosus		2 (3)	2 (7)
Unbalanced atrioventricular septal defect		1 (2)	0
Left-sided heart lesions			
Coarctation of the aorta		9 (15)	4 (14)
Hypoplastic left heart syndrome		4 (7)	1 (3)
Interrupted aortic arch		3 (5)	2 (7)
Hypoplastic aortic arch		2 (3)	0
Aortic stenosis		1 (2)	2 (7)
Right-sided heart lesions			
Tetralogy of Fallot		6 (10)	2 (7)
Pulmonary stenosis		2 (3)	0
Pulmonary atresia		2 (3)	1 (3)
Tricuspid dysplasia		2 (3)	0
Surgical factors			
Cardiac intervention by catheterization, *N* (%)		-	2 (7)
Cardiopulmonary bypass, *N* (%)		-	23 (79)
Time on bypass [mins], median (IQR)		-	143 (122–153)

### Hippocampal volumes in infants with CHD

**Figure 2:**
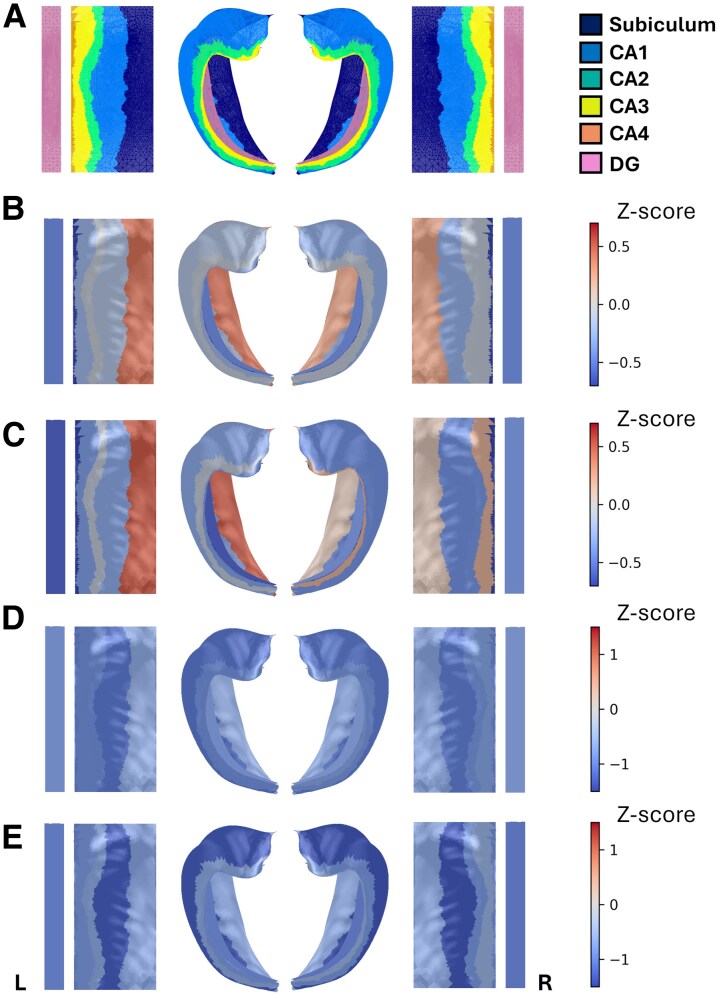
**Hippocampal subfield volume and gyrification Z-scores projected onto folded and unfolded hippocampal surfaces**. **(**A) hippocampal subfields, (B) preoperative volume Z-scores in infants with CHD (*N* = 60), (C) postoperative volume Z-scores in infants with CHD (*N* = 29), (D) preoperative gyrification Z-scores in infants with CHD (*N* = 60) and (E) postoperative gyrification Z-scores in infants with CHD (*N* = 29). (B–E) Warmer colours represent positive Z-scores and cooler colours represent negative Z-scores. Abbreviations: CA, cornus ammonis; CHD, congenital heart disease; DG, dentate gyrus; L, left; R, right.

**Figure 3:**
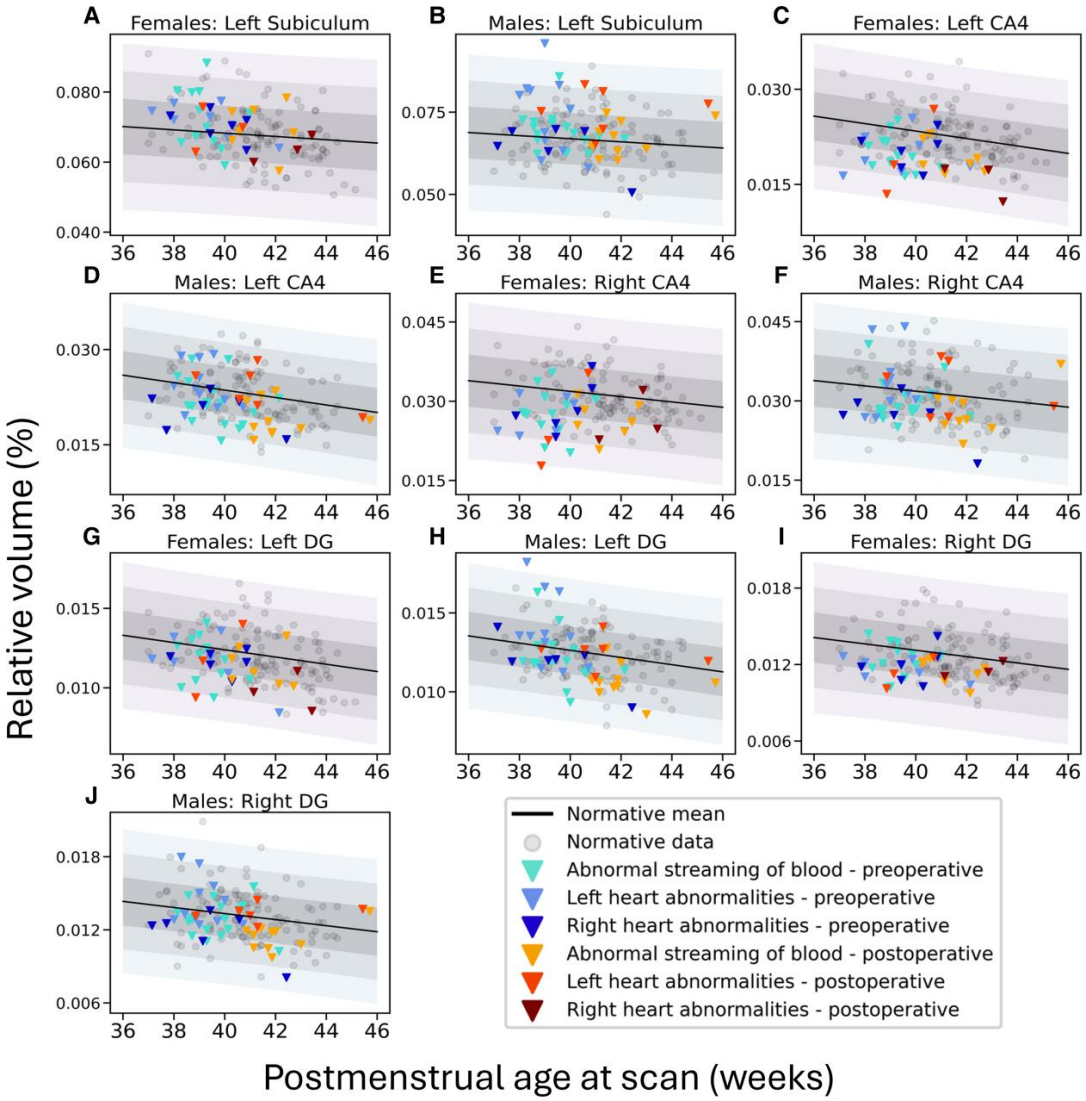
**Relative regional hippocampal volumes in infants with CHD (60 infants with CHD scanned preoperatively, 29 infants with CHD scanned postoperatively) that were significantly different from typically developing infants**. **(A–J)** Relative regional hippocampal volumes in infants with CHD overlaid on the normative model, accounting for postmenstrual age at scan, postnatal age at scan and sex. The normative mean derived from an independent population of 217 healthy control infants is shown as a black line. Shaded areas represent ±1, 2 and 3 standard deviations from the normative model mean, shown separately for female and male infants. Individual data points for typically developing infants are shown in light grey. Data points for infants with CHD are shown as colour triangles (see key in figure). Abbreviations: CA, cornus ammonis; CHD, congenital heart disease; DG, dentate gyrus.

**Figure 4:**
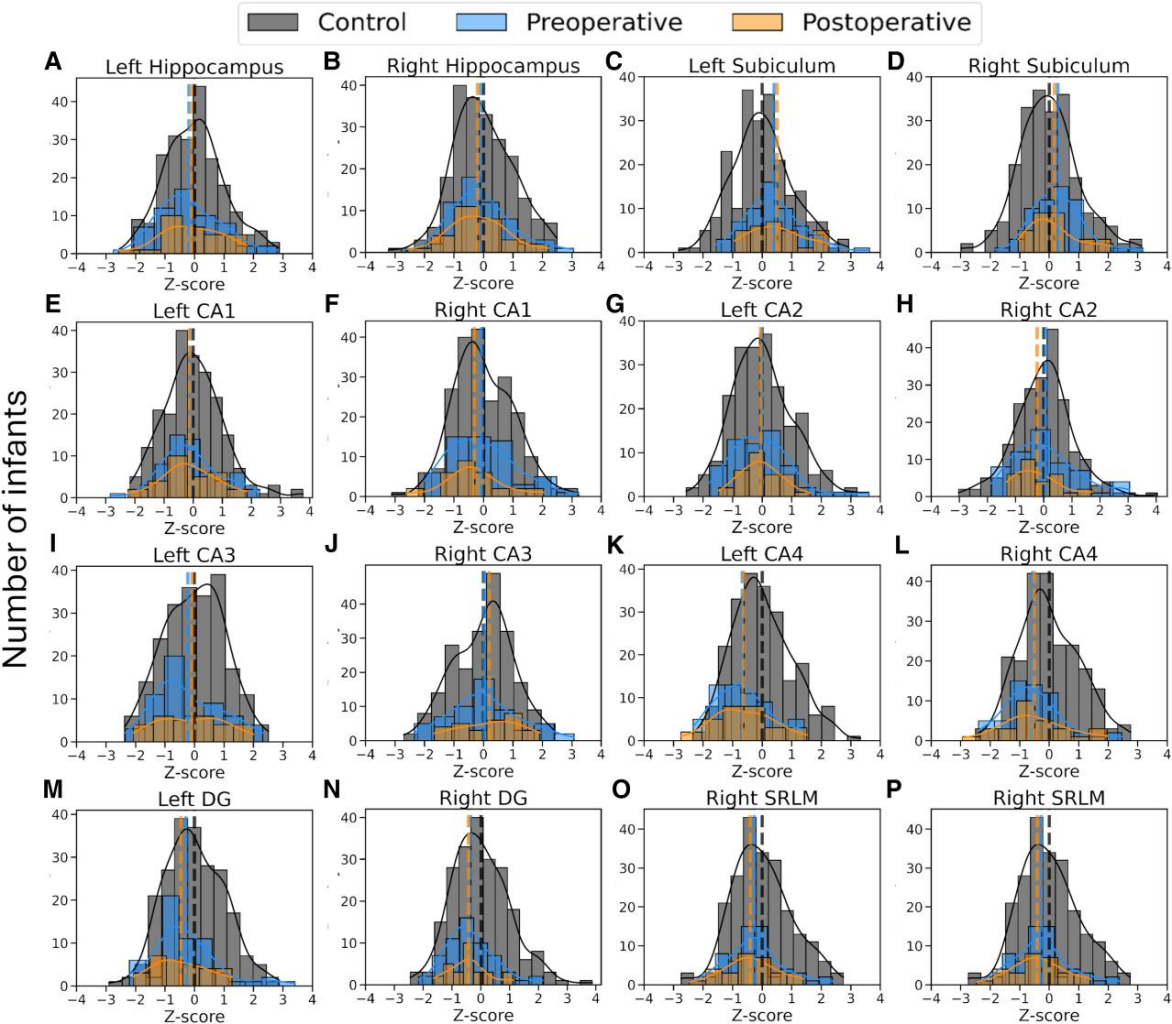
**Relative regional hippocampal volume Z-score histograms**. Histograms showing the number of CHD cases and controls (y axis) versus relative hippocampal volume Z-scores (*x*-axis) for the bilateral (A, B) total hippocampus, (C, D) subiculum, (E, F) CA1, (G, H) CA2, (I, J) CA3, (K, L) CA4, (M, N) DG and (O, P) SRLM for the control cohort (*N* = 217; grey), preoperative CHD (*N* = 60; blue) and postoperative CHD (*N* = 29; orange). Abbreviations: CA, cornus ammonis; CHD, congenital heart disease; DG, dentate gyrus; SRLM, stratum radiatum lacunosum and moleculare.

Average absolute and relative total hippocampal and hippocampal subfield volumes for typically developing infants and infants with CHD are presented in Supplementary Table 1. Table 2 shows the results for differences in relative hippocampal volume Z-scores between the groups. In infants with CHD, relative hippocampal volume Z-scores ranged between −2.85 and 3.63 before surgery and −2.94 and 2.26 after surgery. Preoperative and postoperative relative hippocampal bilateral CA4 and DG volume Z-scores were significantly lower and left subiculum volume Z-scores were significantly higher in infants with CHD, relative to the normative sample (Table 2; Fig. 2B and C). Total hippocampal and all other hippocampal subfield volume Z-scores were not significantly different between infants with CHD and controls. Figure 3 and Supplementary Fig. 1 show total hippocampus, subiculum, CA1, CA2, CA3, CA4, DG and SRLM relative hippocampal volumes for all neonates with CHD overlaid on the normative mean data derived from the independent sample of 217 typically developing infants. Histograms showing the distribution of hippocampal volume Z-scores for the total hippocampus and hippocampal subfield volumes are shown in Fig. 4.

**Table 2:** Relative hippocampal volume Z-scores in infants with CHD compared to normative reference values

Region	Preoperative CHD Z-score (*N* = 60)	pFDR	Postoperative CHD Z-score (*N* = 29)	pFDR
Left hippocampus Mean (±SD)	−0.15 (±1.09)	0.57	−0.10 (±1.01)	0.65
Right hippocampus Mean (±SD)	−0.06 (±1.05)	0.81	−0.12 (±0.99)	0.45
Left subiculum Mean (±SD)	0.44 (±0.99)	**0.02**	0.51 (±0.91)	**0**.**04**
Right subiculum Median (IQR)	0.27 (−0.29 to 0.72)	0.05	0.09 (−0.44 to 0.48)	0.45
Left CA1 Median (IQR)	−0.14 (−0.71 to 0.39)	0.81	−0.30 (−0.53 to 0.54)	0.64
Right CA1 Median (IQR)	−0.26 (−0.99 to 0.70)	0.92	−0.41 (−0.90 to 0.03)	0.27
Left CA2 Median (IQR)	−0.05 (−0.66 to 0.57)	0.96	−0.07 (−0.58 to 0.36)	0.65
Right CA2 Median (IQR)	−0.06 (−0.76 to 0.61)	0.77	−0.39 (−0.89 to 0.21)	0.27
Left CA3 Median (IQR)	−0.22 (−0.96 to 0.62)	0.47	−0.35 (−0.98 to 0.67)	0.69
Right CA3 Mean (±SD)	−0.07 (±1.20)	0.81	0.19 (±1.05)	0.45
Left CA4 Median (IQR)	−0.74 (−1.38 to −0.11)	**<0.001**	−0.82 (−1.3 to 0.05)	**0**.**03**
Right CA4 Median (IQR)	−0.70 (−1.19 to −0.12)	**0**.**01**	−0.73(−1.1 to −0.02)	**0**.**03**
Left DG Median (IQR)	−0.49 (−0.88 to 0.21)	**0**.**02**	−0.64(−1.1 to 0.21)	**0**.**04**
Right DG Median (IQR)	−0.46 (−0.91 to 0.13)	**0**.**03**	−0.45 (−0.81 to −0.22)	**0**.**04**
Left SRLM Median (IQR)	−0.44 (−1.0 to −0.01)	0.05	−0.37 (−0.94 to 0.27)	0.10
Right SRLM Median (IQR)	−0.34 (−0.81 to 0.09)	0.22	−0.40 (−0.86 to 0.07)	0.08

Abbreviations: CA, cornu ammonis; CHD, congenital heart disease; DG, dentate gyrus; FDR, false discovery rate; IQR, interquartile range; SD, standard deviation; SRLM, stratum radiatum lacunosum and moleculare. Results in bold are significant (pFDR < 0.05).

Post hoc analyses revealed bilateral relative CA4 volume Z-scores were significantly reduced preoperatively in infants with abnormal streaming of blood (Supplementary Table 2; Supplementary Fig. 2). Total hippocampal and all other hippocampal subfield volume Z-scores were not significantly different between controls and infants in each CHD category.

### Hippocampal gyrification in infants with CHD

**Figure 5:**
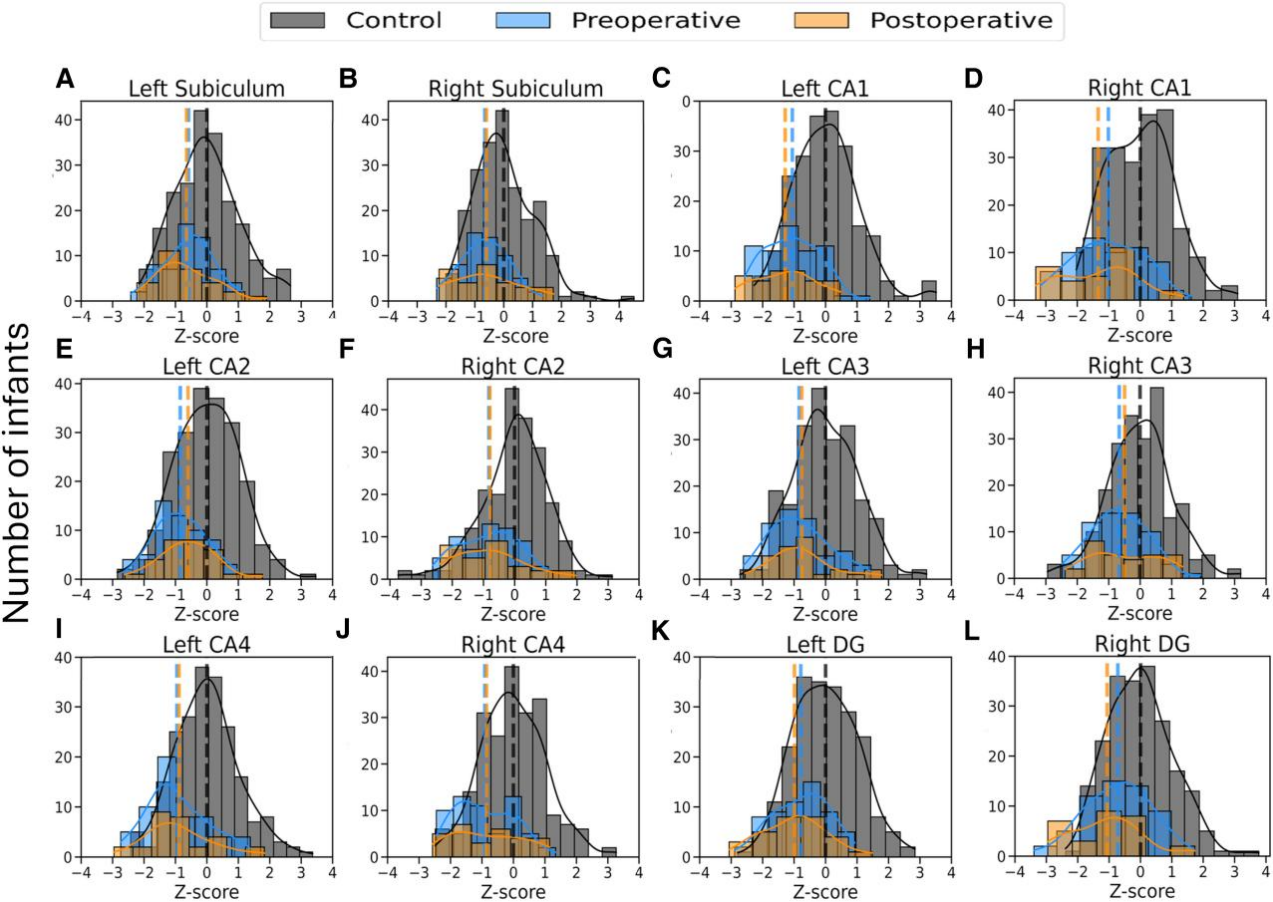
**Hippocampal subfield gyrification Z-score histograms**. Histograms showing the number of CHD cases and controls (y axis) versus hippocampal gyrification Z-scores (*x*-axis) for the bilateral (A, B) subiculum, (C, D) CA1, (E, F) CA2, (G, H) CA3, (I, J) CA4 and (K, L) DG for the control cohort (*N* = 217; grey), preoperative CHD (*N* = 60; blue) and postoperative CHD (*N* = 29; orange). Abbreviations: CA, cornus ammonis; CHD, congenital heart disease; DG, dentate gyrus.

Hippocampal subfield gyrification for typically developing infants and infants with CHD are presented in Supplementary Table 3. In infants with CHD, hippocampal subfield gyrification Z-scores ranged between −3.39 and 1.92 before surgery and −3.32 and 1.90 after surgery. Hippocampal gyrification Z-scores were significantly lower for the bilateral subiculum, CA1, CA2, CA3, CA4 and DG in infants with CHD pre- and postoperatively (Table 3; Fig. 2D and E). Histograms showing the distribution of hippocampal subfield gyrification Z-scores are shown in Fig. 5.

**Table 3:** Hippocampal subfield gyrification Z-scores in infants with CHD compared to normative reference values

Region	Preoperative CHD Z-score (*N* = 60)	pFDR	Postoperative CHD Z-score (*N* = 29)	pFDR
Left subiculum Mean (±SD)	−0.58 (±0.79)	**<0.001**	−0.65 (±0.91)	**0**.**002**
Right subiculum Mean (±SD)	−0.63 (±0.80)	**<0**.**001**	−0.62 (±1.12)	**0**.**008**
Left CA1 Mean (±SD)	−1.06 (±0.87)	**<0**.**001**	−1.28 (±0.94)	**<0**.**001**
Right CA1 Mean (±SD)	−1.01 (±1.09)	**<0**.**001**	−1.33 (±1.19)	**<0**.**001**
Left CA2 Mean (±SD)	−0.85 (±0.79)	**<0**.**001**	−0.60 (±0.88)	**0**.**001**
Right CA2 Mean (±SD)	−0.80 (±0.92)	**<0**.**001**	−0.79 (±1.05)	**<0**.**001**
Left CA3 Mean (±SD)	−0.84 (±0.94)	**<0**.**001**	−0.75 (±0.99)	**<0**.**001**
Right CA3 Mean (±SD)	−0.67 (±0.90)	**<0**.**001**	−0.50 (±1.07)	**0**.**03**
Left CA4 Mean (±SD)	−0.95 (±0.96)	**<0**.**001**	−0.88 (±1.06)	**<0**.**001**
Right CA4 Mean (±SD)	−0.91 (±0.91)	**<0**.**001**	−0.85 (±1.10)	**<0**.**001**
Left DG Mean (±SD)	−0.79 (±0.89)	**<0**.**001**	−0.99 (±1.02)	**<0**.**001**
Right DG Mean (±SD)	−0.72 (±1.05)	**<0**.**001**	−1.06 (±1.15)	**<0**.**001**

Abbreviations: CA, cornu ammonis; CHD, congenital heart disease; DG, dentate gyrus; FDR, false discovery rate; SD, standard deviation. Results in bold are significant (pFDR < 0.05).

Post hoc analyses revealed that preoperative hippocampal gyrification Z-scores were significantly lower for the bilateral subiculum, CA1, CA2, CA3, CA4 and DG in infants with abnormal streaming of blood, left-sided cardiac lesions and right-sided cardiac lesions. Hippocampal gyrification Z-scores were significantly reduced postoperatively for the bilateral subiculum, CA1, CA2, CA3, CA4 and DG in infants with abnormal streaming of blood and for the bilateral CA1 in infants with left-sided cardiac lesions (Supplementary Table 4; Supplementary Fig. 3).

### Sensitivity analyses

The results after removing infants with genetic syndromes are reported in the Supplementary Materials (Supplementary Tables 5 and 6). Hippocampal morphometric Z-scores that were significantly different between controls and all infants with CHD (preoperative = 60, postoperative = 29) remained significant after removing infants with genetic syndromes (preoperative = 3, postoperative = 4).

### Comparison of hippocampal morphometry between infants with cyanotic and acyanotic CHD

Thirty-eight infants had cyanotic CHD (36 preoperative scans, 21 postoperative scans) and 27 infants had acyanotic CHD (24 preoperative scans; 8 postoperative scans). Diagnoses that were not clearly cyanotic or acyanotic were classified based on oxygen saturation levels. Infants with an oxygen saturation below 90% at the time of scanning were considered cyanotic. Cyanotic and acyanotic CHD diagnoses are reported in Supplementary Table 7. Total hippocampus and hippocampal subfield relative volume Z-scores (Supplementary Table 8) and hippocampal gyrification Z-scores (Supplementary Table 9) were not significantly different between infants with cyanotic and acyanotic CHD either before or after surgery.

### Association between hippocampal volume and gyrification Z-scores and CDO_2_ in infants with CHD

No significant associations were identified between CDO_2_ and total hippocampus or hippocampal subfield relative volume Z-scores (Supplementary Table 10) or hippocampal subfield gyrification Z-scores (Supplementary Table 11).

### Neurodevelopmental assessments at 22 months in infants with CHD

Fifty-two infants with CHD attended a follow-up neurodevelopmental assessment at a median (IQR) age of 22.32 (22.06–22.95) months (Table 4). One infant died before 22 months of age, six declined follow-up, and six lived too far away to attend. Cognitive and motor composite scores in infants with CHD who attended the follow-up assessment are summarized in Table 4.

**Table 4:** Neurodevelopmental assessment results for infants with CHD

	Infants with CHD, *N* = 52
Age at neurodevelopmental assessment in months, median (IQR)	22.32 (22.06–22.95)
Cognitive composite score, mean (SD)	93 (±8)
Motor composite score, mean (SD)	96 (±7)

When comparing infants who attended the neurodevelopmental assessment (*N* = 52) and those who did not attend (*N* = 13), there were no significant differences in IMD (W = 326, *P* = 0.93), gestational age at birth (W = 241, *P* = 0.13) or sex (χ^2^ = 0.318, *P* = 0.57).

Neurodevelopmental outcome measures were analysed for 48 infants with CHD scanned preoperatively (three infants only had postoperative data and IMD was not available for one infant). There was no association between preoperative total hippocampus or hippocampal subfield relative volume Z-scores (Supplementary Table 12) or hippocampal subfield gyrification Z-scores (Supplementary Table 13) and cognitive composite scores or motor composite scores, after accounting for IMD.

### Association between hippocampal morphometry Z-scores and socioeconomic status in infants with CHD

IMD was available for 215 typically developing infants, 59 infants with preoperative CHD and 29 infants with postoperative CHD (Supplementary Table 14; Supplementary Fig. 4). There were no significant differences in the distribution of IMD quintiles between infants with preoperative CHD and typically developing infants (χ² = 2.27, *P* = 0.69). There was a significant difference in the distribution of IMD quintiles between infants with postoperative CHD and typically developing controls (*P* = 0.002). No significant associations were identified between IMD and hippocampal morphometric Z-scores (Supplementary Tables 15 and 16).

## Discussion

The hippocampus plays a central role in learning and memory.^36^ Previous studies have identified smaller hippocampal volumes in fetuses,^12^ infants,^5,13^ children^14-16^ and adolescents^17-19^ with CHD and smaller hippocampal volumes have been associated with impaired cognition and executive functioning.^15,17-19^ As such, investigating hippocampal morphometry in early life is important for improving our understanding of brain structural alterations that may contribute to neurodevelopmental impairments in this population. Here, we used a normative modelling approach to characterize individualized measures of total hippocampus and hippocampal subfield volume and gyrification in infants with CHD. Our findings reveal that hippocampal morphometry is altered in these infants before and after surgery; however these alterations are not uniform across the hippocampus.

### Hippocampal volumes are altered in infants with CHD

We found that relative volumes of the bilateral CA4 and DG were reduced, while the relative volume of the left subiculum was increased in infants with CHD compared to typically developing infants. No significant differences were identified in other hippocampal subfield volumes or total hippocampal volumes, after accounting for the total brain size. While previous studies have reported smaller total hippocampal volumes in CHD,^12-19^ our findings are consistent with previous work in infants with CHD which did not observe differences in relative hippocampal volume^13^ and suggests that reductions in total hippocampal volume are proportional to the smaller global brain volumes observed in infants with CHD.^3-5,7,13^

The CA4 and DG are functionally interconnected, receiving neuronal input from the entorhinal cortex and sending neuronal output to the CA3 to support key hippocampal functions such as memory encoding and early recall.^36^ The CA4 and DG subfields have been implicated in cognitive abilities in individuals born prematurely; smaller DG volumes are associated with lower IQ in young adults born before 32 weeks gestation^37^ and smaller CA4/DG volumes are associated with reduced working memory in very low birthweight children,^38^ suggesting that the development of these subfields is important for cognitive abilities across childhood. Interestingly, while smaller CA4/DG volumes were reported in young adults with CHD, no associations between CA4/DG volumes and self-reported measures of working memory ability were identified.^18^

In this study, we detected a relative increase in volume of the left subiculum in infants with CHD. The subiculum receives direct input from the CA1 and serves as a major output structure of the hippocampus, relaying information to cortical and subcortical areas.^36^ Previous studies have reported smaller relative subiculum volumes in young adults with CHD compared to healthy controls.^18,19^ It is possible that the growth trajectory of the subiculum is altered in CHD and further longitudinal studies are required to better understand the development of the subiculum in this population.

### Hippocampal gyrification is reduced in infants with CHD

To our knowledge, this is the first study to report altered hippocampal gyrification in infants with CHD. We observed reduced gyrification across all hippocampal subfields after adjusting for volume.

During prenatal development, the hippocampus undergoes rapid growth, initially forming as a flat tissue that progressively folds onto itself,^39^ resulting in infolding of the hippocampal tissue and deepening of the hippocampal sulcus. By approximately 18–20 weeks gestation, the dentate gyrus and cornus ammonis fold into the temporal lobe, resembling the interlocking ‘C-shape’ of the adult hippocampus.^39,40^ During infancy, the hippocampus undergoes rapid development but this growth begins to slow at approximately 2 years of age.^41^ This developmental window represents a critical period during which adverse developmental conditions may impact hippocampal morphometry. Our finding that hippocampal gyrification is reduced after adjusting for volume suggests that infants with CHD exhibit alterations in hippocampal morphology and not merely a reduction in the hippocampal size. This reduction in gyrification may be specific to the hippocampus as we have shown previously that reductions in cortical gyrification are proportional to reductions in the brain size in this population.^23^ Our novel finding that hippocampal subfield gyrification is reduced in infants with CHD adds to the growing body of research on altered hippocampal development in CHD. Future research assessing survivors of CHD could investigate whether the reduced hippocampal gyrification observed in our study persists into childhood and beyond.

### Hippocampal morphometry is similar in infants with cyanotic and acyanotic CHD but not between CHD categories

We did not identify a significant difference in hippocampal morphometry Z-scores between infants with cyanotic and acyanotic CHD. This finding is in line with prior work that reported reduced hippocampal volumes in adolescents with CHD compared to controls but no significant differences between adolescents with cyanotic and acyanotic CHD after correcting for global brain volume.^17^

When we examined diagnostic categories, preoperative hippocampal gyrification Z-scores were reduced across all subfields in all three CHD subgroups. Postoperatively, infants with abnormal streaming of blood demonstrated decreased gyrification across all subfields. Infants with left-sided cardiac lesions demonstrated significant reductions in hippocampal gyrification only in the bilateral CA1 subfield; however, the sample size was small (*N* = 9) which may have limited our ability to detect differences in this group. In addition, this postoperative left-sided cardiac lesion subgroup was composed mostly of infants with CoA, with only one infant with HLHS. Further research, with larger sample sizes, is required to determine whether infants with postoperative left-sided and right-sided cardiac lesions demonstrate reduced hippocampal gyrification.

### Hippocampal morphometry is not associated with CDO_2_

We did not identify significant correlations between preoperative CDO_2_ and relative hippocampal volume or hippocampal gyrification Z-scores. Prior studies have reported significant associations between reduced CDO_2_ and reduced total brain volumes in third-trimester fetuses^42^ and infants^43^ with CHD. Similarly, we previously reported significant positive correlations between CDO_2_ and preoperative total brain, cortical GM and dGM volumes,^3,5,7^ preoperative cortical gyrification^3^ and isometric brain size^44^ in a heterogeneous cohort of CHD infants. However, in line with the results of this study, other studies report that, after accounting for global brain size, there are no significant associations between CDO_2_ and cortical gyrification^23^ or voxel-wise volumetric differences,^5^ suggesting that reduced CDO_2_ primarily impairs global brain growth.

The complex interaction between genetic and environmental factors are also important influences on hippocampal development in CHD.^45^ In both foetuses with CHD and in healthy foetuses with a family history of CHD, absolute regional brain volumes including the hippocampus were smaller compared to healthy foetuses without a family history of CHD, although relative hippocampal volumes were preserved.^12^ In addition, a study that reported a high prevalence of stress and anxiety in pregnant women carrying foetuses with CHD identified an association between maternal stress and impaired hippocampal development in foetuses with CHD.^46^ These studies suggest that genetic and environmental factors may contribute to hippocampal morphometric alterations in CHD.

### Hippocampal morphometry is not associated with neurodevelopmental outcomes at 22 months

We did not identify any associations between preoperative hippocampal morphometry Z-scores and measures of cognitive ability or motor function at 22 months of age in infants with CHD. Previous work has reported a positive relationship between total hippocampal volumes and full-scale IQ and working memory in children and adolescents with CHD.^14,15,17,19^ It is possible that associations between hippocampal morphometry and neurodevelopmental outcomes may not become evident until a later age. Further longitudinal studies are required to determine whether neonatal hippocampal morphometry relates to IQ or academic attainment in later childhood and beyond.

### Study limitations

Although most infants with abnormal streaming of blood or left-sided cardiac lesions undergo surgery within the first few weeks of life, many infants with right-sided cardiac lesions do not typically undergo surgery until several months of age. Therefore, our sample of infants with right-sided cardiac lesions scanned postoperatively was small.

## Conclusion

This study shows that infants with CHD are at increased risk of altered hippocampal morphometry compared to a large cohort of typically developing infants. However, our findings suggest that altered hippocampal morphometry in infants with CHD is not uniform across the hippocampus and emphasise the importance of studying hippocampal development at the subfield level in CHD populations. We did not identify significant relationships between hippocampal morphometry and preoperative CDO_2_, suggesting that in utero and neonatal hypoxia may not fully explain hippocampal morphometric alterations and there are likely other factors that contribute to altered hippocampal development in infants with CHD. Furthermore, preoperative hippocampal morphometry was not associated with neurodevelopmental outcomes at 22 months in CHD and further longitudinal studies are required to determine whether neonatal hippocampal morphometry is related to neurodevelopmental outcomes in later childhood and beyond.

## Supplementary Material

fcag060_Supplementary_Data

## Data Availability

The data that support the findings of this study are available from the corresponding author upon reasonable request. The GPR code used in this study is freely available on GitHub (https://github.com/ralidimitrova/GPR_NeoVols).
