# Supplementary material for: Hippocampal morphometry is altered in infants with congenital heart disease
Source: Brain Commun. 2026 Feb 26;8(2):fcag060. doi: 10.1093/braincomms/fcag060 (PMC12965832; doi:10.1093/braincomms/fcag060)
Supplement: fcag060_Supplementary_Data [file fcag060_supplementary_data.pdf]

Supplementary Materials

Hippocampal morphometry is altered in infants with congenital heart disease

Barat Gal-Er[1], Alexandra F. Bonthrone[1], Emily S. Nichols[2,3], Andrew T.M. Chew[1], Daniel Cromb[1], Chiara Casella[1,4], Alexia Egloff[1], Kuberan Pushparajah[5,6], John Simpson[5,6], Mary A. Rutherford[1], A. David Edwards[1], Joseph V. Hajnal[1,7], Chiara Nosarti[1,8], Jonathan O'Muircheartaigh[1,4], Emma G. Duerden[2,3], Serena J. Counsell[1]

1. Centre for the Developing Brain, Research Department of Early Life Imaging, School of Biomedical Engineering and Imaging Sciences, King's College London, London, UK

2. Department of Applied Psychology, Faculty of Education, Western University, London, Ontario, Canada

3. Western Institute for Neuroscience, Western University, London, Ontario, Canada

4. Department for Forensic and Neurodevelopmental Sciences, Institute of Psychiatry, Psychology and Neuroscience, King's College London, London, UK

5. Department of Cardiovascular Imaging, School of Biomedical Engineering and Imaging Sciences, King's College London, London, UK

6. Department of Fetal and Paediatric Cardiology, Evelina London Children's Hospital, London, UK

7. Research Department of Imaging Physics & Engineering, School of Biomedical Engineering and Imaging Sciences, King's College London, London, UK

8. Department of Child and Adolescent Psychiatry, Institute of Psychiatry, Psychology and Neuroscience, King's College London, London, UK

Supplementary Table 1. Perioperative absolute and relative hippocampal volumes in infants with CHD.

Region	Median absolute volume (IQR) [mm^3]			Median relative volume (IQR) [%]		
	Controls (N=217)	Preoperative (N=60)	Postoperative (N=29)	Controls (N=217)	Preoperative (N=60)	Postoperative (N=29)
Left hippocampus	1111 (1016 -1197)	966 (870 - 1049)	960 (874 - 1084)	0.33 (0.31 - 0.36)	0.33 (0.32 - 0.37)	0.33 (0.30 - 0.36)
Right hippocampus	1167 (1071 -1279)	1018 (921 - 1120)	1015 (886 - 1142)	0.36 (0.33 - 0.38)	0.36 (0.33 - 0.38)	0.35 (0.32 - 0.36)
Left subiculum	239.3 (215.8 -266.5)	215.6 (203.0 -233.9)	213.4 (195.6 -243.1)	0.073 (0.07 - 0.08)	0.077 (0.07 - 0.08)	0.075 (0.07 - 0.08)
Right subiculum	245.7 (220.2 -273.9)	219.2 (203.0 -241.6)	218.0 (198.7 -252.5)	0.075 (0.07 - 0.08)	0.078 (0.07 - 0.08)	0.077 (0.07 – 0.08)
Left CA1	301.0 (279.9 -328.1)	272.1 (244.2 -294.0)	268.6 (235.7 -292.4)	0.092 (0.09 - 0.10)	0.096 (0.09 - 0.10)	0.092 (0.08 - 0.1)
Right CA1	333.8 (307.2 -361.9)	293.2 (268.6 -326.6)	294.8 (255.7 -311.3)	0.10 (0.09 - 0.11)	0.10 (0.1 - 0.11)	0.10 (0.09 - 0.10)
Left CA2	57.2 (47.1 - 65.8)	49.2 (42.5 - 56.0)	48.7 (42.8 - 54.0)	0.017 (0.015 - 0.02)	0.017 (0.015 - 0.02)	0.017 (0.015 - 0.02)
Right CA2	62.6 (53.2 - 70.4)	53.2 (44.3 - 60.7)	50.4 (41.7 - 58.1)	0.019 (0.016 - 0.02)	0.018 (0.015 - 0.02)	0.017 (0.015 - 0.02)
Left CA3	155.1 (132.8 -179.2)	120.4 (106.1 -152.8)	135.0 (111.5 -147.0)	0.048 (0.04 - 0.05)	0.043 (0.04 - 0.05)	0.044 (0.04 - 0.05)
Right CA3	123.2 (103.1 -140.3)	103.6 (88.6 - 123.6)	109.7 (88.1 - 138.1)	0.038 (0.03 - 0.04)	0.037 (0.03 - 0.04)	0.040 (0.03 - 0.04)
Left CA4	80.8 (71.3 - 92.2)	64.9 (57.7 - 73.8)	62.35 (54.3 - 65.3)	0.025 (0.02 - 0.03)	0.023 (0.02 - 0.03)	0.020 (0.02 - 0.03)
Right CA4	110.5 (97.0 - 125.7)	89.6 (76.8 - 98.7)	88.2 (75.4 - 101.5)	0.033 (0.03 - 0.04)	0.031 (0.03 - 0.034)	0.029 (0.027 - 0.03)
Left DG	43.7 (39.6 - 48.4)	37.2 (33.5 - 41.7)	34.9 (33.0 - 37.4)	0.013 (0.01 - 0.02)	0.013 (0.01 - 0.01)	0.012 (0.01 - 0.01)
Right DG	46.0 (41.5 - 51.5)	38.1 (34.6 - 43.8)	38.1 (33.8 - 42.7)	0.014 (0.01 - 0.02)	0.014 (0.01 - 0.02)	0.013 (0.01 - 0.01)
Left SRLM	231.8 (213.8 -253.3)	198.0 (176.5 -215.5)	195.4 (177.6 -214.2)	0.071 (0.07 - 0.08)	0.069 (0.06 - 0.08)	0.068 (0.06 - 0.07)
Right SRLM	248.9 (225.6 -273.4)	212.6 (184.3 -235.0)	211.1 (184.8 -239.0)	0.075 (0.07 - 0.08)	0.075 (0.07 - 0.08)	0.072 (0.07 - 0.08)

Abbreviations: CA, cornu ammonis; CHD, congenital heart disease; DG, dentate gyrus; IQR, interquartile range; SRLM, stratum radiatum lacunosum and moleculare.

Supplementary Table 2. Relative hippocampal volume Z-Scores in infants with abnormal streaming of blood, left-sided cardiac lesions and right-sided cardiac lesions compared to normative reference values.

Region	Preoperative CHD						Postoperative CHD			
	Abnormal streaming of blood Z-score, n =29		Left-sided cardiac lesion Z-score, n=19		Right-sided cardiac lesion Z-score, n=12		Abnormal streaming of blood Z-score, n=17		Left-sided cardiac lesion Z-score, n=9	
	Median (IQR)	pFDR	Median (IQR)	pFDR	Median (IQR)	pFDR	Median (IQR)	pFDR	Median (IQR)	pFDR
Left hippocampus	-0.43 (-0.93 to 0.61)	0.54	-0.17 (-0.62 to 0.97)	0.82	-0.41 (-0.98 to 0.14)	0.23	-0.42 (-0.95 to 0.22)	0.41	0.5 (0.10 to 0.70)	0.28
Right hippocampus	-0.07 (-0.55 to 0.6)	0.93	-0.33 (-0.71 to 0.82)	0.80	-0.44 (-1.0 to -0.08)	0.21	-0.48 (-0.81 to 0.17)	0.54	0.21 (-0.16 to 0.54)	0.97
Left subiculum	0.30 (-0.23 to 0.76)	0.13	0.6 (-0.12 to 1.47)	0.07	0.2 (-0.47 to 0.41)	0.99	0.36 (-0.16 to 1.02)	0.28	0.99 (0.37 to 1.89)	0.10
Right subiculum	0.19 (-0.93 to 0.61)	0.41	0.68 (-0.62 to 0.97)	0.06	0.03 (-0.98 to 0.14)	0.99	-0.18 (-0.95 to 0.22)	0.75	0.17 (0.27 to 0.85)	0.68
Left CA1	0.01 (-0.23 to 0.60)	0.93	-0.14 (-0.12 to 1.0)	0.86	-0.35 (-0.47 to 0.41)	0.54	-0.30 (-0.16 to 1.0)	0.75	0.03 (0.37 to 1.01)	0.80
Right CA1	-0.04 (-0.55 to 0.70)	0.90	-0.2 (-1.08 to 0.82)	0.86	-0.65 (-1.1 to 0.03)	0.21	-0.41 (-0.77 to 0.1)	0.68	-0.4 (-0.52 to 0.03)	0.75
Left CA2	-0.38 (-0.23 to 0.76)	0.80	0.05 (-0.65 to 0.67)	0.90	0.15 (-0.60 to 0.67)	0.86	-0.35 (-0.64 to -0.07)	0.06	0.37 (0.03 to 0.7)	0.75
Right CA2	0.07 (-0.55 to 0.74)	0.64	-0.29 (-1.0 to 0.82)	0.80	0.05 (-1.13 to 0.03)	0.86	-0.36 (-0.77 to 0.10)	0.43	-0.39 (-0.52 to 0.03)	0.68
Left CA3	-0.61 (-0.89 to 0.38)	0.41	-0.27 (-0.76 to 1.21)	0.86	-0.60 (-1.20 to 0.61)	0.54	-0.46 (-0.98 to 0.51)	0.52	0.50 (-0.41 to 0.84)	0.75
Right CA3	-0.05 (-0.44 to 0.36)	0.86	0.04 (-0.70 to 0.77)	0.80	-0.27 (-1.36 to 0.96)	0.84	0.11 (-0.56 to 0.52)	0.97	0.73 (0.30 to 0.90)	0.78
Left CA4	-0.90 (-1.89 to 0.38	**0.01**	-0.35 (-0.76 to 1.21)	0.60	-0.82 (-1.26 to 0.61)	0.05	-0.97 (-0.98 to 0.51)	**0.04**	-0.05 (-0.40 to 0.80)	0.90
Right CA4	-0.71 (-1.07 to -0.15)	**0.03**	-0.19 (-1.16 to 0.26)	0.80	-0.91 (-1.25 to -0.51)	0.07	-0.73 (-0.56 to 0.40)	**0.04**	0.20 (-0.90 to 0.86)	0.81
Left DG	-0.57 (-0.99 to -0.02)	0.07	0.20 (-0.83 to 0.61)	0.80	-0.54 (-0.74 to -0.17)	0.07	-0.84 (-1.13 to -0.02)	0.06	0.21 (-0.53 to 0.78)	0.96
Right DG	-0.38 (-1.07 to -0.15)	0.10	-0.38 (-1.16 to 0.26)	0.85	-0.82 (-1.25 to -0.51)	0.07	-0.49 (-0.56 to 0.52)	0.06	-0.13 (-0.90 to 0.86)	0.96
Left SRLM	-0.40 (-1.02 to 0.10)	0.07	-0.31 (-0.67 to 0.82)	0.94	-0.60 (-1.10 to -0.28)	0.06	-0.50 (-1.01 to -0.11)	0.06	0.42 (0.20 to 0.80)	0.81
Right SRLM	-0.34 (-1.07 to 0.15)	0.57	-0.03 (-1.16 to 0.26)	0.93	-0.64 (-1.25 to -0.51)	0.07	-0.63 (-0.56 to 0.52)	0.08	0.15 (-0.9 to 0.86)	0.97

Abbreviations: CA, cornu ammonis; CHD, congenital heart disease; DG, dentate gyrus; IQR, interquartile range; SRLM, stratum radiatum lacunosum and moleculare.

Supplementary Table 3. Hippocampal gyrification in infants with CHD.

Region	Gyrification index, mean (±SD)		
	Controls (N=217)	Perioperative CHD (N=60)	Postoperative CHD (N=29)
Left subiculum	1.14 (±0.14)	1.05 (±0.12)	1.06 (±0.14)
Right subiculum	1.15 (±0.15)	1.05 (±0.12)	1.06 (±0.17)
Left CA1	1.27 (±0.12)	1.14 (±0.11)	1.11 (±0.12)
Right CA1	1.31 (±0.12)	1.19 (±0.14)	1.14 (±0.15)
Left CA2	0.74 (±0.11)	0.65 (±0.09)	0.66 (±0.10)
Right CA2	0.80 (±0.12)	0.71 (±0.11)	0.71 (±0.13)
Left CA3	1.16 (±0.16)	1.02 (±0.15)	1.04 (±0.19)
Right CA3	1.14 (±0.16)	1.03 (±0.15)	1.06 (±0.18)
Left CA4	1.96 (±0.22)	1.74 (±0.22)	1.76 (±0.25)
Right CA4	2.11 (±0.24)	1.88 (±0.25)	1.91 (±0.29)
Left DG	3.61 (±0.51)	3.21 (±0.45)	3.11 (±0.52)
Right DG	3.78 (±0.51)	3.41 (±0.53)	3.23 (±0.59)

Abbreviations: CA, cornu ammonis; CHD, congenital heart disease; DG, dentate gyrus; SD, standard deviation; SRLM, stratum radiatum lacunosum and moleculare.

Supplementary Table 4. Hippocampal gyrification Z-scores in infants with abnormal streaming of blood, left-sided cardiac lesions and right-sided cardiac lesions compared to normative reference values.

Region	Preoperative CHD						Postoperative CHD			
	Abnormal streaming of blood Z-score, n =29		Left-sided cardiac lesion Z-score, n=19		Right-sided cardiac lesion Z-score, n=12		Abnormal streaming of blood Z-score, n=17		Left-sided cardiac lesion Z-score, n=9	
	Median (IQR)	pFDR	Median (IQR)	pFDR	Median (IQR)	pFDR	Median (IQR)	pFDR	Median (IQR)	pFDR
Left subiculum	-0.42 (-0.94 to 0.09)	**0.03**	-0.68 (-1.09 to -0.28)	**0.04**	-0.76 (-1.31 to -0.21)	**0.003**	-0.52 (-0.96 to -0.08)	**0.04**	-1.21 (-1.91 to -0.46)	0.10
Right subiculum	-0.49 (-0.99 to 0.01)	**0.01**	-0.59 (-1.10 to 0.01)	**0.03**	-0.84 (-1.33 to -0.34)	**0.004**	-0.70 (-1.21 to -0.22)	**0.02**	-1.3 (-2.45 to -0.04)	0.07
Left CA1	-0.92 (-1.56 to -0.28)	**<0.001**	-1.16 (-1.91 to -0.33)	**<0.001**	-1.39 (-1.71 to 0.80)	**<0.001**	-1.08 (-1.52 to -0.56)	**<0.001**	-1.61 (-2.60 to -0.63)	**0.01**
Right CA1	-0.88 (-1.75 to -0.02)	**<0.001**	-1.07 (-2.01 to -0.13)	**0.004**	-1.31 (-1.76 to -0.87)	**<0.001**	-0.83 (-1.68 to 0.01)	**0.001**	-2.33 (-3.48 to -1.10)	**0.009**
Left CA2	-0.77 (-1.19 to -0.35)	**<0.001**	-0.73 (-1.46 to -0.20)	**0.006**	-0.97 (-1.57 to 0.4)	**0.004**	-0.65 (-1.10 to -0.20)	**0.001**	-0.72 (-1.58 to 0.20)	0.35
Right CA2	-0.54 (-1.12 to -0.04)	**0.002**	-1.15 (-1.95 to - 0.22)	**0.001**	-0.69 (-1.33 to -0.10)	**0.002**	-0.64 (-1.15 to -0.10)	**0.003**	-1.32 (-2.22 to -0.41)	0.09
Left CA3	-1.01 (-1.50 to -0.52)	**<0.001**	-0.66 (-1.44 to 0.11)	**0.04**	-1.26 (-2.12 to -0.40)	**0.004**	-1.07 (-1.57 to -0.57)	**0.001**	-0.64 (-1.63 to -0.65)	0.32
Right CA3	-0.49 (-1.03 to -0.8)	**0.002**	-0.88 (-1.53 to -0.20)	**0.03**	-1.12 (-2.01 to -0.23)	**0.013**	-0.64 (-1.43 to 0.15)	**0.04**	-0.11 (-1.31 to 1.08)	0.43
Left CA4	-1.21 (-1.71 to -0.74)	**<0.001**	-0.78 (-1.54 to -0.02)	**0.03**	-1.40 (-1.90 to -0.20)	**0.004**	-0.97 (-1.37 to -0.57)	**<0.001**	-0.19 (-1.41 to 1.04)	0.42
Right CA4	-1.03 (-1.68 to -0.38)	**<0.001**	-0.99 (-1.71 to -0.27)	**0.006**	-1.52 (-2.33 to 0.70)	**0.004**	-1.09 (-1.6 to - 0.42)	**0.001**	-1.24 (-2.30 to -0.17)	0.10
Left DG	-0.75 (-1.26 to -0.24)	**<0.001**	-0.37 (-1.15 to 0.30)	**0.01**	-0.78 (-1.43 to -0.14)	**0.009**	-0.82 (-1.15 to -0.35)	**0.001**	-0.92 (-1.94 to 0.10)	0.17
Right DG	-0.72 (-1.55 to 0.13)	**0.004**	-0.61 (-1.46 to 0.09)	**0.03**	-0.91 (-1.45 to -0.38)	**0.02**	-0.95 (-1.56 to -0.34)	**0.001**	-1.09 (-2.1 to - 0.08)	0.08

Abbreviations: CA, cornu ammonis; CHD, congenital heart disease; DG, dentate gyrus; IQR, interquartile range.

Supplementary Table 5. Relative hippocampal volume Z-Scores in infants with CHD without genetic syndromes compared to normative reference values.

Region	Preoperative CHD Z-score, N=57	pFDR	Postoperative CHD Z-score, N=25	pFDR
Left hippocampus<br>Mean (SD)	-0.19<br>(±1.10)	0.41	-0.16<br>(1.01)	0.54
Right hippocampus<br>Mean (SD)	-0.07<br>(±1.10)	0.85	-0.16<br>(1.04)	0.54
Left subiculum<br>Mean (SD)	0.40<br>(±0.88)	**0.02**	0.50<br>(0.86)	**0.04**
Right subiculum<br>Median (IQR)	0.28<br>(-0.26 to 0.70)	0.06	0.17<br>(-0.28 to 0.60)	0.47
Left CA1<br>Median (IQR)	-0.15<br>(-0.70 to 0.40)	0.55	-0.30<br>(-0.53 to 0.54)	0.54
Right CA1<br>Median (IQR)	-0.05<br>(-0.59 to 0.49)	0.86	-0.25<br>(-0.70 to 0.05)	0.47
Left CA2<br>Median (IQR)	0.05<br>(-0.58 to 0.68)	0.88	-0.11<br>(-0.47 to 0.25)	0.54
Right CA2<br>Median (IQR)	-0.01<br>(-0.71 to 0.69)	0.85	-0.30<br>(-0.89 to 0.28)	0.42
Left CA3<br>Median (IQR)	-0.19<br>(-0.76 to 0.37)	0.46	-0.22<br>(-0.74 to 0.3)	0.50
Right CA3<br>Mean (SD)	0.04<br>(±1.20)	0.68	0.15<br>(±1.12)	0.54
Left CA4<br>Median (IQR)	-0.70<br>(-1.37 to -0.05)	**<0.001**	-0.82<br>(-1.51 to -0.14)	**0.01**
Right CA4<br>Median (IQR)	-0.69<br>(-1.19 to -0.14)	**0.004**	-0.55<br>(-0.94 to 0.27)	**0.04**
Left DG<br>Median (IQR)	-0.49<br>(-1.04 to 0.07)	**0.04**	-0.46<br>(-0.80 to -0.21)	**0.04**
Right DG<br>Median (IQR)	-0.46<br>(-1.03 to 0.11)	**0.03**	-0.45<br>(-0.99 to 0.06)	**0.04**
Left SRLM<br>Median (IQR)	-0.30<br>(-0.75 to 0.20)	0.05	-0.30<br>(-0.70 to 0.30)	0.13
Right SRLM<br>Median (IQR)	-0.11<br>(-0.76 to 0.53)	0.24	-0.35<br>(-0.80 to 0.39)	0.54

Abbreviations: CA, cornu ammonis; CHD, congenital heart disease; DG, dentate gyrus; IQR, interquartile range; SD, standard deviation; SRLM, stratum radiatum lacunosum and moleculare.

Supplementary Table 6. Hippocampal gyrification Z-Scores in infants with CHD without genetic syndromes compared to normative reference values.

Region	Preoperative CHD Z-score, N=57	pFDR	Postoperative CHD Z-score, N=25	pFDR
Left subiculum	-0.51 (±0.94)	**<0.001**	-0.73 (±0.99)	**0.01**
Right subiculum	-0.56 (±1.09)	**<0.001**	-0.70 (±0.97)	**0.04**
Left CA1	-1.04 (±1.03)	**<0.001**	-1.08 (±1.02)	**<0.001**
Right CA1	-0.98 (±0.98)	**<0.001**	-0.84 (±1.09)	**<0.001**
Left CA2	-0.82 (±0.81)	**<0.001**	-0.55 (±0.93)	**0.02**
Right CA2	-0.71 (±0.88)	**<0.001**	-0.64 (±0.99)	**0.004**
Left CA3	-0.82 (±0.96)	**<0.001**	-0.73 (±1.05)	**0.004**
Right CA3	-0.64 (±0.92)	**<0.001**	-0.50 (±1.00)	**0.01**
Left CA4	-0.92 (±0.97)	**<0.001**	-0.77 (±1.10)	**0.002**
Right CA4	-0.87 (±0.91)	**<0.001**	-0.69 (±1.11)	**0.01**
Left DG	-0.75 (±0.85)	**<0.001**	-0.82 (±0.94)	**0.001**
Right DG	-0.67 (±1.03)	**<0.001**	-0.95	**0.001**

Abbreviations: CA, cornu ammonis; CHD, congenital heart disease; DG, dentate gyrus; SD, standard deviation.

Supplementary Table 7. Cyanotic and acyanotic CHD diagnoses.

	Preoperative CHD, N=60	Postoperative CHD, N=29
**Cyanotic CHD, N (%)**		
Transposition of the great arteries	20 (33)	15 (52)
Hypoplastic left heart syndrome	4 (7)	1 (3)
Total anomalous pulmonary venous drainage	3 (5)	0
Tetralogy of Fallot	3 (5)	1 (3)
Truncus arteriosus	2 (3)	2 (7)
Pulmonary atresia	2 (3)	1 (3)
Double outlet right ventricle	1 (2)	0
Interrupted aortic arch	1 (2)	1 (3)
**Acyanotic CHD, N (%)**		
Coarctation of the aorta	9 (15)	4 (14)
Tetralogy of Fallot	3 (5)	1 (3)
Tricuspid dysplasia	2 (3)	0
Pulmonary stenosis	2 (3)	0
Interrupted aortic arch	2 (3)	1 (3)
Double outlet right ventricle	2 (3)	0
Hypoplastic aortic arch	2 (3)	0
Unbalanced atrioventricular septal defect	1 (2)	0
Aortic stenosis	1 (2)	2 (7)

Supplementary Table 8. Relative hippocampal volume Z-Scores in infants with cyanotic CHD compared to infants with acyanotic CHD, separately for preoperative and postoperative CHD.

Hippocampal volume Z-score	Preoperative CHD			Postoperative CHD		
	Cyanotic CHD Median (IQR)	Acyanotic CHD Median (IQR)	pFDR	Cyanotic CHD Median (IQR)	Acyanotic CHD Median (IQR)	pFDR
Left hippocampus	-0.20 (-0.63 to 0.64)	-0.52 (-0.99 to 0.05)	0.48	-0.31 (-0.85 to 0.23)	0.75 (-0.78 to 1.06)	0.70
Right hippocampus	-0.05 (-0.44 to 0.6)	-0.40 (-1.1 to 0.07)	0.17	-0.43 (-0.81 to 0.44)	0.02 (-0.86 to 0.52)	0.97
Left Subiculum	0.28 (-0.18 to 0.79)	0.38 (-0.23 to 1.02)	0.92	0.36 (-0.17 to 1.02)	0.73 (0.27 to 1.28)	0.70
Right subiculum	0.29 (-0.15 to 0.80)	0.34 (-0.20 to 0.90)	0.92	-0.07 (-0.44 to 0.48)	0.13 (-0.35 to 0.39)	0.96
Left CA1	0.09 (-0.56 to 0.89)	-0.52 (-0.78 to -0.11)	0.15	-0.30 (-0.53 to 0.37)	-0.32 (-0.62 to 0.76)	0.98
Right CA1	0.22 (-0.5 to 0.86)	-0.49 (-1.17 to 0.02)	0.15	-0.30 (-0.77 to 0.10)	-0.47 (-1.02 to -0.30)	0.43
Left CA2	-0.01 (-0.65 to 0.6)	0.01 (-0.64 to 0.24)	0.59	-0.18 (-0.58 to 0.17)	0.22 (-0.19 to 0.73))	0.70
Right CA2	0.28 (-0.16 to 0.99)	-0.39 (-0.88 to 0.16)	0.15	-0.36 (-0.84 to 0.27)	-0.64 (-1.04 to 0.17)	0.73
Left CA3	-0.37 (-0.84 to 0.65)	-0.69 (-1.26 to 0.65)	0.59	-0.35 (-0.95 to 0.53)	0.09 (-1.33 to 0.93)	0.97
Right CA3	0.28 (-0.16 to 0.99)	-0.19 (-0.60 to 0.11)	0.49	0.14 (-0.64 to 0.92)	0.24 (-0.54 to 0.93)	0.97
Left CA4	-0.62 (-0.99 to 0.16)	-0.67 (-0.95 to 0.20)	0.98	-0.97 (-1.26 to -0.21)	0.10 (-1.67 to 0.90)	0.70
Right CA4	-0.52 (-0.87 to 0.11)	-0.50 (-0.88 to 0.25)	0.98	-0.76 (-1.09 to -0.10)	0.30 (-1.14 to 0.99)	0.70
Left DG	-0.55 (-0.97 to 0.13)	-0.43 (-0.83 to 0.39)	0.59	-0.79 (-1.13 to 0.05)	-0.3 (-1.1 to 0.9)	0.75
Right DG	-0.38 (-0.93 to 0.24)	-0.54 (-0.87 to -0.11)	0.92	-0.46 (-0.81 to -0.37)	-0.18 (-0.53 to 0.11)	0.70
Left SRLM	-0.27 (-0.85 to 0.02)	-0.62 (-1.18 to 0.06)	0.54	-0.40 (-0.80 to -0.06)	0.37 (-0.9 to 0.92)	0.70
Right SRLM	-0.19 (-0.64 to 0.11)	-0.42 (-1.34 to -0.03)	0.45	-0.58 (-0.86 to -0.05)	0.32 (-0.89 to 0.73)	0.70

Abbreviations: CA, cornu ammonis; CHD, congenital heart disease; DG, dentate gyrus; IQR, interquartile range; SRLM, stratum radiatum lacunosum and moleculare.

Supplementary Table 9. Hippocampal gyrification Z-Scores in infants with cyanotic CHD compared to infants with acyanotic CHD, separately for preoperative and postoperative CHD.

Hippocampal gyrification Z-score	Preoperative CHD			Postoperative CHD		
	Cyanotic CHD Mean (SD)	Acyanotic CHD Mean (SD)	pFDR	Cyanotic CHD Mean (SD)	Acyanotic CHD Mean (SD)	pFDR
Left subiculum	-0.65 (±0.82)	-0.48 (±0.73)	0.86	-0.75 (±0.81)	-0.38 (±1.15)	0.87
Right subiculum	-0.74 (±0.80)	-0.46 (±0.80)	0.77	-0.61 (±1.03)	-0.63 (±1.41)	0.97
Left CA1	-1.07 (±0.78)	-1.04 (±1.00)	0.95	-1.32 (±1.05)	-1.2 (±1.29)	0.97
Right CA1	-1.0 (±0.93)	-1.02 (±1.30)	0.95	-1.32 (±1.05)	-1.39 (±1.62)	0.97
Left CA2	-0.83 (±0.68)	-0.86 (±0.95)	0.95	-0.69 (±0.56)	-0.36 (±1.47)	0.66
Right CA2	-0.68 (±0.88)	-0.99 (±0.97)	0.77	-0.85 (±0.87)	-0.65 (±1.49)	0.73
Left CA3	-0.88 (±0.77)	-0.79 (±1.16)	0.95	-1.01 (±1.00)	-0.55 (±1.10)	0.60
Right CA3	-0.62 (±0.68)	-0.73 (±1.17)	0.95	-0.50 (±0.69)	0.47 (±1.54)	0.95
Left CA4	-1.12 (±0.73)	-0.71 (±1.20)	0.77	-0.99 (±0.65)	-0.61 (±1.79)	0.56
Right CA4	-1.03 (±0.81)	-0.74 (±1.05)	0.77	-0.91 (±1.03)	-0.75 (±1.36)	0.66
Left DG	-0.87 (±0.88)	-0.67 (±0.91)	0.85	-1.05 (±0.82)	-0.84 (±1.46)	0.95
Right DG	-0.77 (±0.97)	-0.65 (±1.17)	0.95	-1.17 (±0.88)	-0.77 (±1.17)	0.73

Abbreviations: CA, cornu ammonis; CHD, congenital heart disease; DG, dentate gyrus; SD, standard deviation.

Supplementary Table 10. The relationship between cerebral oxygen delivery and hippocampal volume Z-scores.

Hippocampal volume Z-score	Correlation coefficient (pFDR)
Left hippocampus	R^2 = -0.05 (0.68)
Right hippocampus	R^2 = -0.10 (0.42)
Left subiculum	R^2 = -0.10 (0.46)
Right subiculum	ρ = -0.04 (0.80)
Left CA1	ρ = -0.08 (0.56)
Right CA1	ρ = -0.02 (0.86)
Left CA2	ρ = 0.008 (0.95)
Right CA2	ρ = -0.10 (0.10)
Left CA3	ρ = 0.03 (0.81)
Right CA3	R^2 = -0.02 (0.87)
Left CA4	ρ = -0.01 (0.93)
Right CA4	ρ = 0.01 (0.93)
Left DG	ρ = 0.14 (0.30)
Right DG	ρ = 0.10 (0.46)
Left SRLM	ρ = -0.05 (0.67)
Right SRLM	ρ = -0.09 (0.48)

Abbreviations: CA, cornu ammonis; DG, dentate gyrus; SRLM, stratum radiatum lacunosum and moleculare.

Supplementary Table 11. The relationship between cerebral oxygen delivery and hippocampal gyrification Z-scores.

Hippocampal gyrification Z-score	Pearson's R^2 (pFDR)
Left subiculum	-0.05 (0.72)
Right subiculum	-0.005 (0.96)
Left CA1	-0.02 (0.86)
Right CA1	-0.05 (0.73)
Left CA2	0.09 (0.48)
Right CA2	-0.15 (0.20)
Left CA3	0.11 (0.44)
Right CA3	-0.08 (0.52)
Left CA4	0.16 (0.25)
Right CA4	-0.03 (0.77)
Left DG	0.02 (0.86)
Right DG	-0.06 (0.64)

Abbreviations: CA, cornu ammonis; DG, dentate gyrus.

Supplementary Table 12. Partial Spearman's rank correlations between preoperative relative hippocampal volume Z-scores and cognitive and motor outcomes, adjusting for IMD.

Hippocampal volume Z-score	Spearman's coefficient, ρ (pFDR)	
	Cognitive composite score	Motor composite score
Left hippocampus	0.13 (0.97)	0.19 (0.74)
Right hippocampus	-0.02 (0.97)	0.07 (0.74)
Left subiculum	-0.12 (0.97)	-0.18 (0.74)
Right subiculum	-0.05 (0.97)	-0.07 (0.74)
Left CA1	0.09 (0.97)	0.10 (0.74)
Right CA1	-0.01 (0.97)	0.05 (0.76)
Left CA2	0.09 (0.97)	-0.07 (0.74)
Right CA2	-0.17 (0.56)	-0.10 (0.74)
Left CA3	0.07 (0.97)	0.10 (0.74)
Right CA3	-0.06 (0.97)	-0.10 (0.74)
Left CA4	0.02 (0.97)	0.16 (0.74)
Right CA4	0.02 (0.90)	0.11 (0.74)
Left DG	0.21 (0.56)	0.37 (0.16)
Right DG	0.10 (0.97)	0.05 (0.85)
Left SRLM	0.11 (0.56)	0.23 (0.70)
Right SRLM	0.01 (0.99)	0.17 (0.74)

Abbreviations: CA, cornu ammonis; DG, dentate gyrus; SRLM, stratum radiatum lacunosum and moleculare.

Supplementary Table 13. Partial Spearman's rank correlations between preoperative hippocampal gyrification Z-scores and cognitive and motor outcomes, adjusting for IMD.

Hippocampal gyrification Z-score	Spearman's coefficient, ρ (pFDR)	
	Cognitive composite score	Motor composite score
Left subiculum	0.03 (0.84)	0.20 (0.65)
Right subiculum	-0.08 (0.84)	0.14 (0.65)
Left CA1	0.03 (0.84)	0.14 (0.65)
Right CA1	-0.04 (0.84)	0.12 (0.65)
Left CA2	-0.06 (0.84)	-0.05 (0.88)
Right CA2	-0.02 (0.84)	-0.10 (0.65)
Left CA3	-0.04 (0.84)	0.05 (0.88)
Right CA3	-0.10 (0.84)	-0.12 (0.65)
Left CA4	0.08 (0.84)	0.19 (0.65)
Right CA4	0.11 (0.84)	0.04 (0.88)
Left DG	0.04 (0.84)	0.20 (0.65)
Right DG	0.10 (0.85)	0.02 (0.88)

Abbreviations: CA, cornu ammonis; DG, dentate gyrus.

Supplementary Table 14. Index of Multiple Deprivation (IMD) quintiles in controls and infants with CHD.

Index of Multiple Deprivation quintile, N (%)	Controls (N=217)	Preoperative CHD (N=60)	Postoperative CHD (N=29)
First (most deprived)	27 (12)	9 (15)	6 (21)
Second	89 (41)	14 (23)	4 (14)
Third	49 (23)	15 (25)	5 (17)
Fourth	31 (14)	10 (17)	6 (21)
Fifth (least deprived)	19 (9)	11 (18)	8 (28)
Missing	2 (1)	1 (2)	0 (0)

Supplementary Table 15. The relationship between IMD and hippocampal volume Z-scores.

Hippocampal volume Z-score	Spearman's coefficient, ρ (pFDR)	
	Preoperative CHD, N=59	Postoperative CHD, N=29
Left hippocampus	-0.17(0.43)	-0.31 (0.06)
Right hippocampus	-0.12 (0.65)	-0.23 (0.33)
Left subiculum	-0.07 (0.79)	-0.32 (0.06)
Right subiculum	-0.02 (0.92)	-0.15 (0.43)
Left CA1	-0.03 (0.92	-0.23 (0.33)
Right CA1	-0.06 (0.79)	-0.22 (0.35)
Left CA2	-0.01 (0.92)	-0.20(0.36)
Right CA2	-0.07 (0.79)	-0.27 (0.33)
Left CA3	-0.18 (0.43)	-0.36 (0.06)
Right CA3	-0.14 (0.61)	-0.15 (0.44)
Left CA4	-0.21 (0.43)	-0.32 (0.24)
Right CA4	-0.19 (0.43)	-0.16 (0.44)
Left DG	-0.23 (0.43)	-0.38 (0.06)
Right DG	-0.20 (0.43)	-0.24 (0.33)
Left SRLM	-0.20 (0.43)	-0.35 (0.06)
Right SRLM	-0.10 (0.70)	-0.23 (0.33)

Abbreviations: CA, cornu ammonis; CHD, congenital heart disease; DG, dentate gyrus; SRLM, stratum radiatum lacunosum and moleculare.

Supplementary Table 16. The relationship between IMD and hippocampal gyrification Z-scores.

Gyrification Z-score	Spearman's coefficient, ρ (pFDR)	
	Preoperative CHD, N=59	Postoperative CHD, N=29
Left subiculum	-0.01 (0.98)	0.23 (0.22)
Right subiculum	0.002 (0.99)	0.35 (0.26)
Left CA1	-0.05 (0.99)	0.30 (0.34)
Right CA1	0.01 (0.99)	0.38 (0.27)
Left CA2	-0.16 (0.75)	0.16 (0.57)
Right CA2	-0.02 (0.99)	0.07 (0.70)
Left CA3	-0.19 (0.76)	-0.08 (0.70)
Right CA3	-0.12 (0.75)	0.18 (0.57)
Left CA4	-0.2 (0.75)	0.15 (0.57)
Right CA4	-0.15 (0.75)	0.21 (0.52)
Left DG	-0.15 (0.76)	0.13 (0.62)
Right DG	0.09 (0.86)	0.36 (0.26)

Abbreviations: CA, cornu ammonis; CHD, congenital heart disease; DG, dentate gyrus.

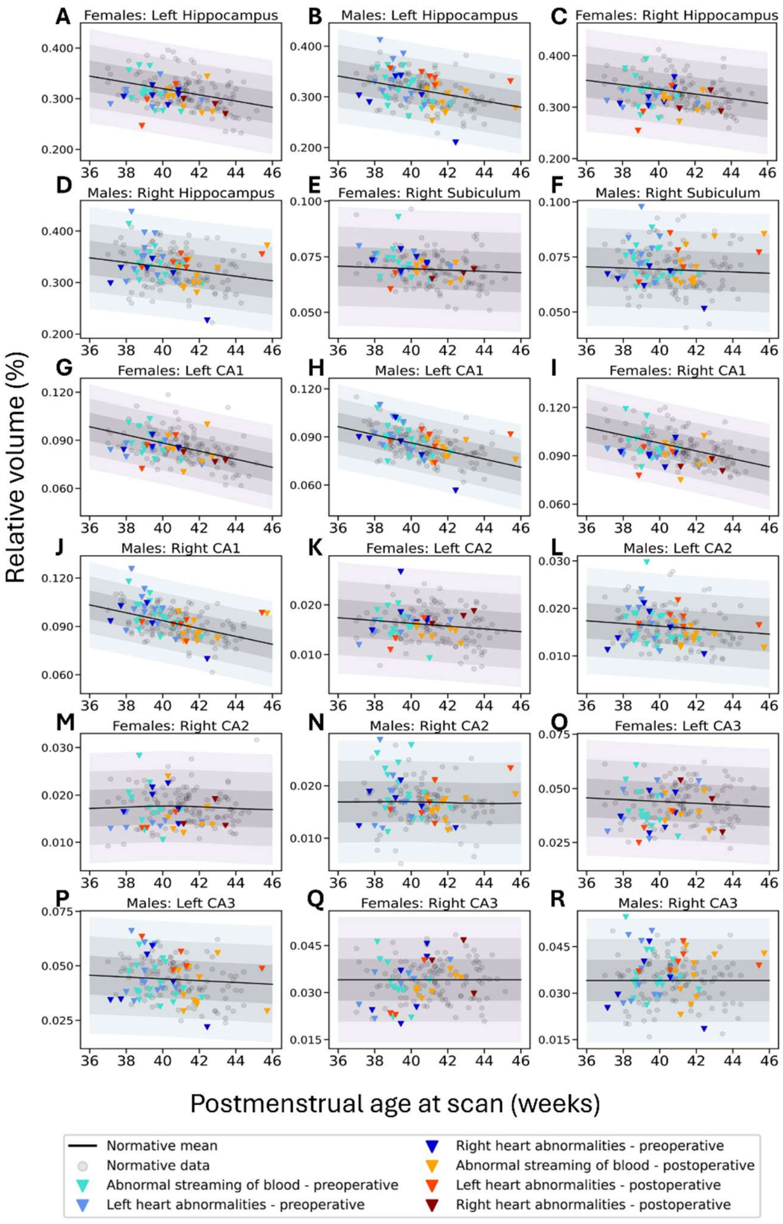

Supplementary Figure 1. Relative regional hippocampal volumes in infants with CHD (60 infants with CHD preoperatively, 29 infants with CHD postoperatively) that were not significantly different from typically developing infants. A-R) Relative regional hippocampal volumes in infants with CHD overlaid on the normative model, accounting for postmenstrual age at scan, postnatal age at scan and sex. The normative mean derived from an independent population of 217 healthy control infants is shown as a black line. Shaded areas represent ±1, 2, and 3 standard deviations from the normative model mean, shown separately for female and male infants. Individual data points for typically developing infants are shown in

light grey. Data points for infants with CHD are shown as colour triangles (see key in figure). Abbreviations: CA, cornus ammonis; CHD, congenital heart disease; DG, dentate gyrus.

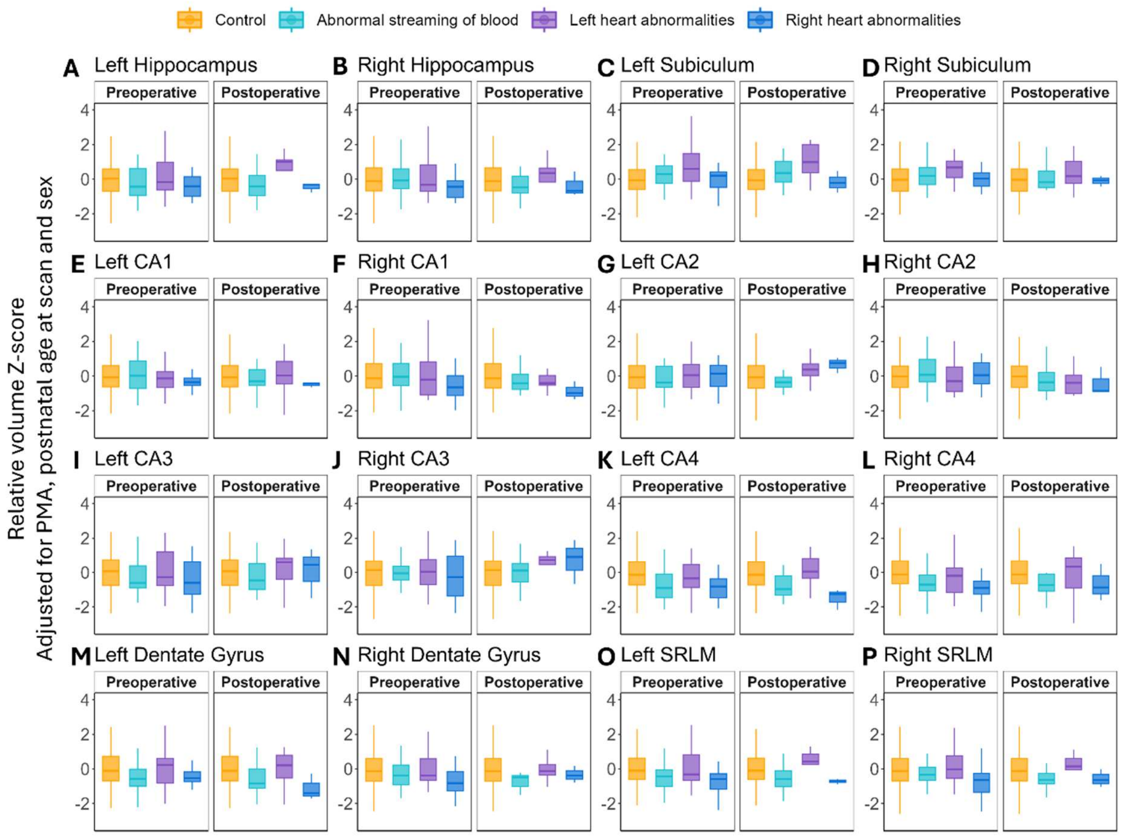

Supplementary Figure 2. A-P) Boxplots showing relative hippocampal volume Z-scores for typically developing infants (orange) and three categories of infants with CHD preoperatively (abnormal streaming, N = 29; left-sided cardiac lesions, N = 19; right-sided cardiac lesions, N = 12) and postoperatively (abnormal streaming, N = 17; left-sided cardiac lesions, N = 9; right-sided cardiac lesions, N = 3). Boxes represent the interquartile range (25th to 75th percentiles), the horizontal line inside the box denotes the median and the vertical line indicates the data range. Abbreviations: CA, cornus ammonis; DG, dentate gyrus; PMA, postmenstrual age.

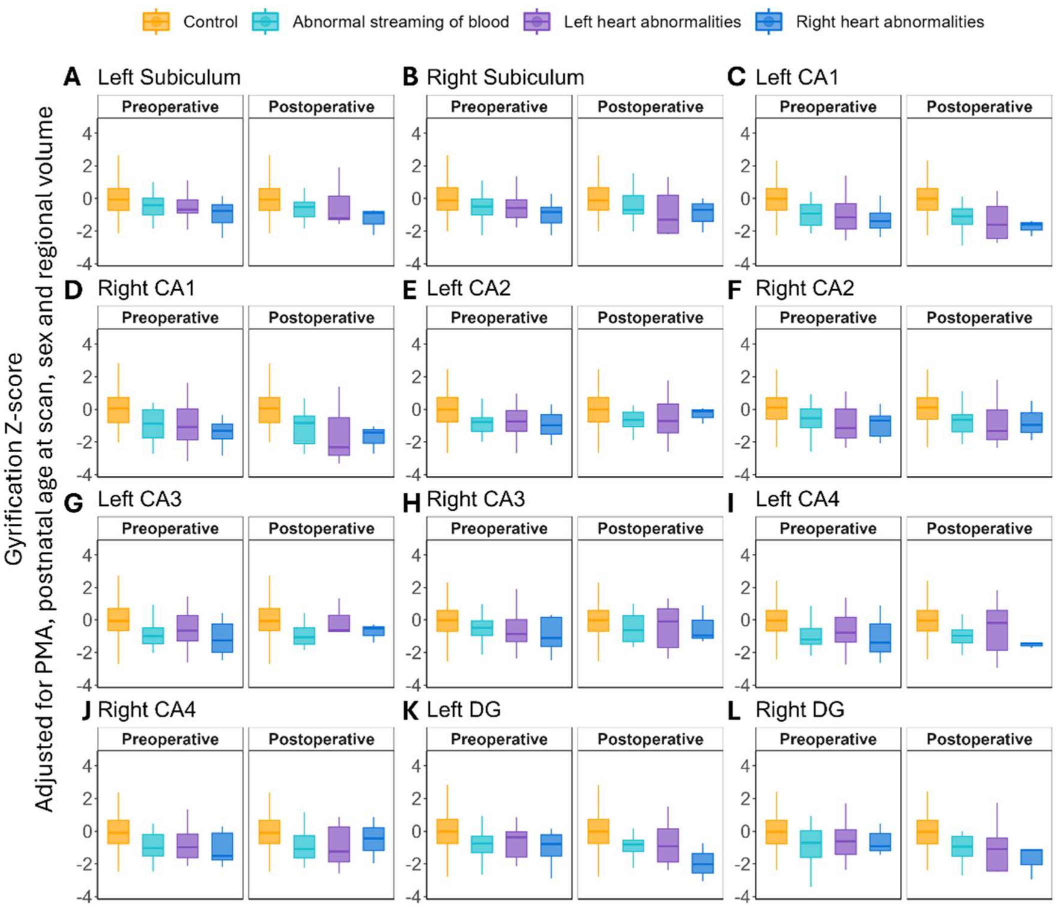

Supplementary Figure 3. A-L) Boxplots showing hippocampal gyrification Z-scores for typically developing infants (orange) and three categories of infants with CHD preoperatively (abnormal streaming, N = 29; left-sided cardiac lesions, N = 19; right-sided cardiac lesions, N = 12) and postoperatively (abnormal streaming, N = 17; left-sided cardiac lesions, N = 9; right-sided cardiac lesions, N = 3). Boxes represent the interquartile range (25th to 75th percentiles), the horizontal line inside the box denotes the median and the vertical line indicates the data range. Abbreviations: CA, cornus ammonis; DG, dentate gyrus; PMA, postmenstrual age.

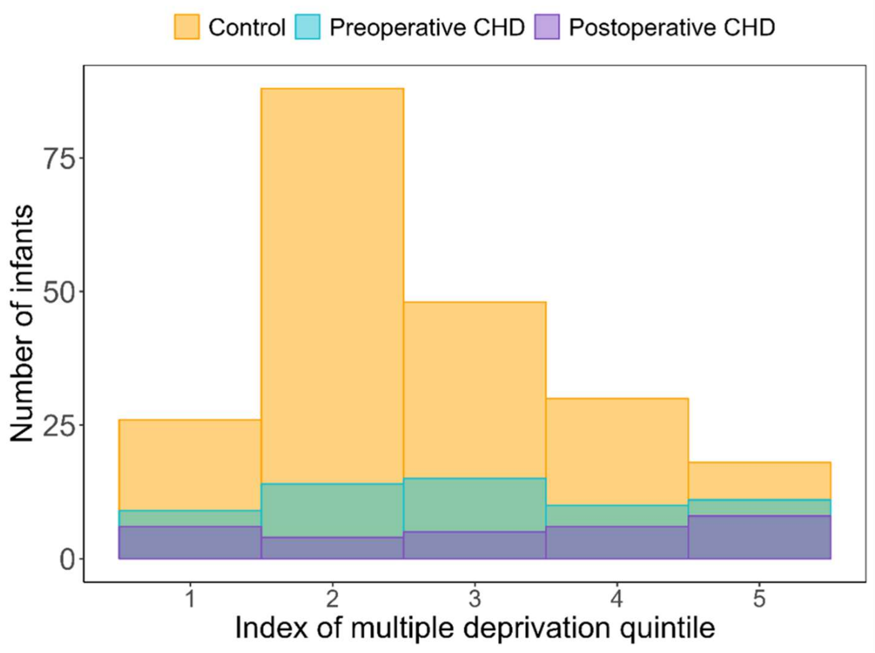

Supplementary Figure 4. Histogram of index of multiple deprivation (IMD) quintiles in controls (orange), infants with CHD scanned preoperatively (blue) and infants with CHD scanned postoperatively (purple).
